# Tuning Selectivity in the Direct Conversion of Methane to Methanol: Bimetallic Synergistic Effects on the Cleavage of C–H and O–H Bonds over NiCu/CeO_2_ Catalysts

**DOI:** 10.1021/acs.jpclett.2c00885

**Published:** 2022-06-14

**Authors:** Pablo G. Lustemberg, Sanjaya D. Senanayake, José A. Rodriguez, M. Verónica Ganduglia-Pirovano

**Affiliations:** † Instituto de Catálisis y Petroleoquímica, CSIC, C/Marie Curie 2, 28049 Madrid, Spain; ‡ Instituto de Fisica Rosario (IFIR), CONICET-UNR, Bv. 27 de Febrero 210bis, 2000EZP Rosario, Santa Fe, Argentina; § Chemistry Division, Brookhaven National Laboratory, Upton, New York 11973, United States

## Abstract

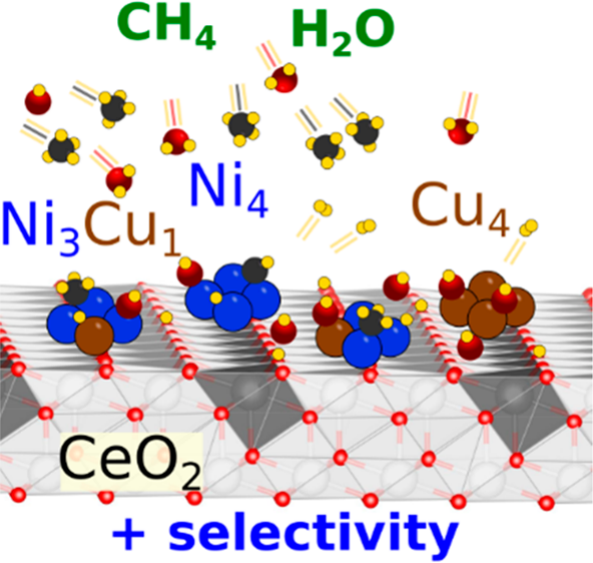

The efficient activation of methane and the simultaneous water dissociation are crucial in many catalytic reactions on oxide-supported transition metal catalysts. On very low-loaded Ni/CeO_2_ surfaces, methane easily fully decomposes, CH_4_ → C + 4H, and water dissociates, H_2_O→ OH + H. However, in important reactions such as the direct oxidation of methane to methanol (MTM), where complex interplay exists between reactants (CH_4_, O_2_), it is desirable to avoid the complete dehydrogenation of methane to carbon. Remarkably, the barrier for the activation of C–H bonds in CH_*x*_ (*x* = 1–3) species on Ni/CeO_2_ surfaces can be manipulated by adding Cu, forming bimetallic NiCu clusters, whereas the ease for cleavage of O–H bonds in water is not affected by ensemble effects, as obtained from density functional theory-based calculations. CH_4_ activation occurs only on Ni sites, and H_2_O activation occurs on both Ni and Cu sites. The MTM reaction pathway for the example of the Ni_3_Cu_1_/CeO_2_ model catalyst predicts a higher selectivity and a lower activation barrier for methanol production, compared with that for Ni_4_/CeO_2_. These findings point toward a possible strategy to design active and stable catalysts which can be employed for methane activation and conversions.

Methane is the main component of natural gas and a major challenge when emitted into the atmosphere because of its contribution to greenhouse effects.^1−3^ Its activation is challenging because of the high C–H bond strength,^4−6^ but its conversion is of great importance for the synthesis of fuels and chemicals while leading to major environmental benefits.^2,3^ Also, water dissociation is an important parallel reaction step in numerous catalytic reactions on oxide-supported transition-metal catalysts, such as the water-gas shift reaction^7−11^ and the conversion of methane by steam reforming.^12−14^ Moreover, the role of water in the direct catalytic conversion of methane or CO_2_ to methanol has recently been highlighted.^15^ Therefore, being able to control the ability of catalysts to simultaneously activate methane and dissociate water is highly desirable. However, this is a challenging task, particularly for oxide-supported metal nanoparticles.^16^ This is because the activity for C–H and O–H bond breaking can be affected by many factors such as the nature of both the metal and the oxide support, the metal particle size and composition, as well as metal–support interactions.

It has been shown that small Ni nanoparticles on a (111)-oriented ceria model support promote the activation of both O–H and C–H bonds in H_2_O and CH_4_, respectively, at room temperature, with lower activation barriers than for extended metallic Ni surfaces.^17−21^ Studies with other metals such as Pt, Co, and Cu indicated that Pt and Co are also active for C–H bond activation, whereas Cu is not.^18,22^

In the case of low-loaded Ni/CeO_2_ catalysts, we have recently shown that the activation of the first C–H bond from CH_4_ to yield CH_3_ is facile and also that the adsorbed CH_4_ undergoes full decomposition to produce C atoms.^12,15,17,20^ Both components of the Ni–ceria interface probably participate in the removal of hydrogen from methane to generate carbon. The rapid deactivation through carbon deposition on high-loaded metal-based catalysts is well-known,^17,18,23,24^ but this is not the case for low-loaded catalysts. In this regard, for low-loaded Ni/CeO_2_ catalysts, no CH_*x*_ or C species were observed under methane dry- or steam-reforming conditions (*T* > 550 K).^12,17^ In the context of the steam reforming, the OH-mediated carbon removal has been discussed.^12,25^ Moreover, in connection with the direct oxidation of methane to methanol (MTM), water was found to play a crucial role in blocking catalytic sites where methyl species could fully decompose, an essential factor for diminishing the production of CO and CO_2_, and in generating sites on which methoxy CH_3_O species and ultimately methanol can form and desorb.^15^ However, is there another strategy to achieve a similar control of the reaction selectivity by minimizing the decomposition of CH_*x*_ and CH_3_O intermediates?

In the field of heterogeneous catalysis by metals, bimetallic catalysts are promising for performing challenging chemical transformations,^26−31^ and the ensemble effect is a well-known phenomenon.^32^ The main feature of this effect is that reactions are sensitive to alloying with a second metal within the group of adjacent atoms of the active metal that are required for forming chemisorption complexes. The effects of alloying may be rationalized by a combination of geometric and electronic effects, the separation of which is not always straightforward. The concept of geometric ensemble implies that the metal atoms in the surface of the alloy keep their individuality and are only influenced by their immediate environment. Electronic effects in alloy catalysts refer to the formation of a bond between two different metals that can induce substantial electronic perturbations leading to changes in the chemical and catalytic properties of the bonded metals.^26−31^ In this context, it is important to establish if bimetallic nickel catalysts, such as NiCu/CeO_2_, can be useful for tuning the degree of dehydrogenation of CH_*x*_ (*x* = 1–3) species on Ni/CeO_2_ surfaces, which is critical for preventing coke formation and crucial in the direct partial oxidation of methane to methanol or in the conversion of CH_*x*_ groups into high-value chemicals like olefins or aromatics.

In this work, we use density functional theory-based calculations within the DFT+U approach, including long-range dispersion corrections, to study in detail the binding and dehydrogenation of methane, as well as the dissociation of water on Ni_4–*x*_Cu_*x*_/CeO_2_(111) bimetallic surfaces with five different concentration ratios of 0%, 25%, 50%, 75%, and 100% (hereinafter referred to as Ni_4–*x*_Cu_*x*_.CeO_2_). In addition, the complete reaction pathway for the direct oxidation of methane to methanol was calculated for the example of Ni_3_Cu_1_/CeO_2_(111) and compared to that for Ni_4_/CeO_2_(111). Our results reveal that by adding Cu to low-loaded Ni/CeO_2_ catalysts, the activation energy barrier for the cleavage of C–H bonds in methyl CH_3_ species significantly increases and that a small percentage of Cu is already sufficient for the effect to be noticeable, whereas it does not appreciably affect the ability of the system to active O–H bonds in water. The results further suggest that, on NiCu bimetallic catalysts, the formation of methoxy species from methyl and oxygen species is favored over further dehydrogenation of the methyl species and that the activation energy for the formation of methanol from methoxy and H species is smaller compared with monometallic Ni catalysts.

The models used are derived from the stable planar rhombohedral Ni_4_ cluster on CeO_2_(111)^20,33,34^ by replacing one, two, and up to the four Ni atoms by Cu, as reported by Salcedo et al.^35^ (see Figure 1; for details on the models, stability, and computational methods, see the Supporting Information). These Ni_4–*x*_Cu_*x*_ clusters are compact but allow a study of the electronic and ensemble effects present in NiCu/CeO_2_ bimetallic systems.^35^ Moreover, the planar Ni_4_.CeO_2_ model mimics the essential features of the experimental Ni/CeO_2_ catalysts for the direct conversion of methane to methanol (Ni^δ+^/CeO_2_),^15^ and in previous works,^17,22,36^ we have investigated such ceria-supported small metallic (Ni, Co, Pt) clusters as theoretical model catalysts in close collaboration with studies employing experimental model catalysts, as well as real (powder) systems for reactions such as the dry-reforming of methane.

**Figure 1:**
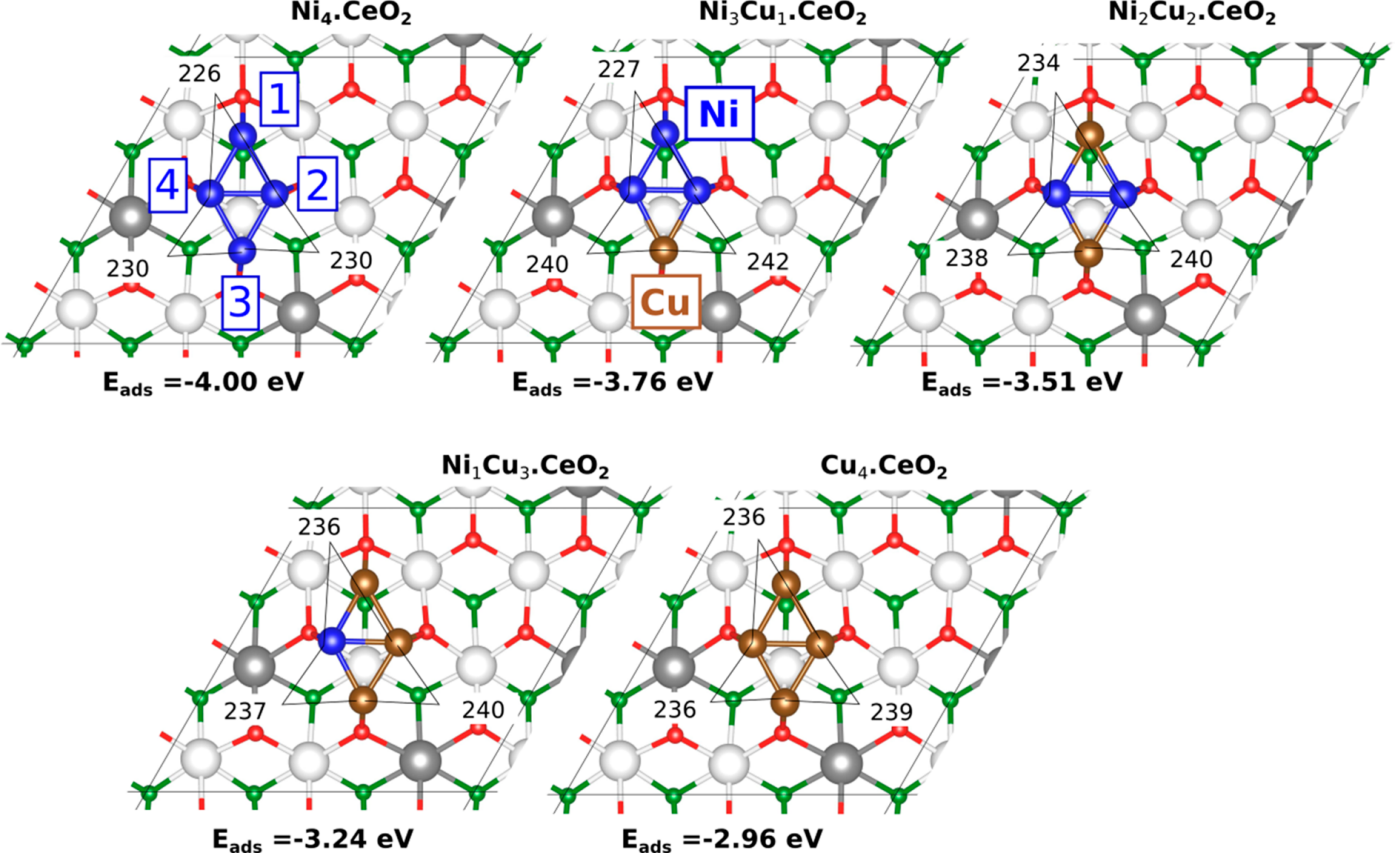
Adsorbed Ni_4–*x*_Cu_*x*_ (*x* = 0 to 4) clusters on CeO_2_(111). Ni and Cu atoms are depicted in blue and brown, respectively, while surface/subsurface oxygen atoms are in red/green, Ce^4+^ in white, and Ce^3+^ in gray. Selected interatomic distances (in pm) are indicated. The average adsorption energy for each model is indicated (with respect to the isolated Cu_gas_ and Ni_gas_ atoms, in eV). The four metal atoms are labeled according to their positions with respect to the ceria surface as 1 to 4.

The Ni_4–*x*_Cu_*x*_.CeO_2_ systems involve Ni and Cu species with similar electron donor–electron acceptor properties, and the flow of charge is here influenced by the existence of the Ni–Cu bond in the bimetallic clusters, the geometrical arrangement of the atoms, and the existence of the ceria support. As a result of strong metal–support interactions, all clusters reduce the ceria support with the formation of two Ce^3+^ (Figure 1). The calculated Bader charges indicate that all atoms in the ceria-supported Ni_4–*x*_Cu_*x*_ (*x* = 0 to 4) clusters lose charge (i.e., Ni_4 – *x*_^δ+^Cu_*x*_^δ+^.CeO_2_, Table S1). However, the atoms that lie along the longer diagonal (atoms 1 and 3, Figure 1), coordinated to two other metal atoms, transfer more charge than those that lie along the shorter diagonal (atoms 2 and 4), which are surrounded by three metal atoms. This is in line with the flow of charge observed for the gas-phase clusters, which result from the removal of the CeO_2_(111) support from the Ni_4–*x*_Cu_*x*_.CeO_2_ systems, without further optimization of the geometry (hereinafter referred to as Ni_4–*x*_Cu_*x*_.gas, Table S1), according to which, the atoms 2 and 4 in 3-fold coordination, give charge to the atoms 1 and 3 in 2-fold coordination. Therefore, it could be summarized by saying that the atoms with the most charge in the gas phase are the ones that transfer the most charge to the ceria support. Furthermore, we also examined the effect of forming bimetallic clusters on the d-projected density of states of the metal atoms for the supported and gas-phase clusters (Table S2). As expected, an increase in the percentage of occupied Ni d-states in the Ni_4–*x*_Cu_*x*_.CeO_2_ and Ni_4–*x*_Cu_*x*_.gas systems (*x* = 1–3), compared with Ni_4_.CeO_2_ and Ni_4_.gas, respectively, is observed. In the case of the occupied Cu d-states, the effect is the reverse with an upward shift of the center of the atom-project *d*-band relative to the Fermi level.

The most stable configurations of the CeO_2_(111)-supported Ni_4–*x*_Cu_*x*_ (*x* = 0 to 4) clusters (Figures 1 and S1) were used to study the activation of both CH_4_ and H_2_O.

In the following, we will discuss the first hydrogen abstraction from CH_4_, i.e., CH_4_ → CH_3_+H, first for the monometallic Ni_4_.CeO_2_ and Cu_4_.CeO_2_ surfaces, as compared with Ni and Cu(111), respectively. The dissociation of CH_4_ is slightly exothermic with Δ*E*= −0.09 eV over the extended Ni(111) surface, whereas over Cu(111) is endothermic with Δ*E*= +0.64 eV (Table S3, Figures S2 and S3). Moreover, the first C–H bond cleavage over these surfaces is very difficult at low temperatures, because of large energy barriers of 0.90 and 1.42 eV for the Ni and Cu surfaces, respectively (Figures 2b and S2).^18,22^ This is in clear contrast with the results obtained for small monometallic Ni_4_ and Cu_4_ particles supported on CeO_2_(111), for which the reaction is highly exothermic with Δ*E* = −0.8 and −1.05 eV (Figure 2a, Table S3), respectively. In addition, the reduction of the energy barriers, compared to the corresponding extended (111) metal surfaces, is noticeably, particularly for the case of the Ni_4_ particles (Figure 2a). The barrier over Ni_4_.CeO_2_ is 0.14 eV, whereas that over Cu_4_.CeO_2_ is 1.08 eV. This is in line with the results of experiments that show that methane dissociates on low-loaded Ni/CeO_2_ at temperatures as low as 300 K, generating CH_*x*_ and CO_*x*_ species on the catalyst surface, whereas Cu/CeO_2_ has a negligible activity.^18^

**Figure 2:**
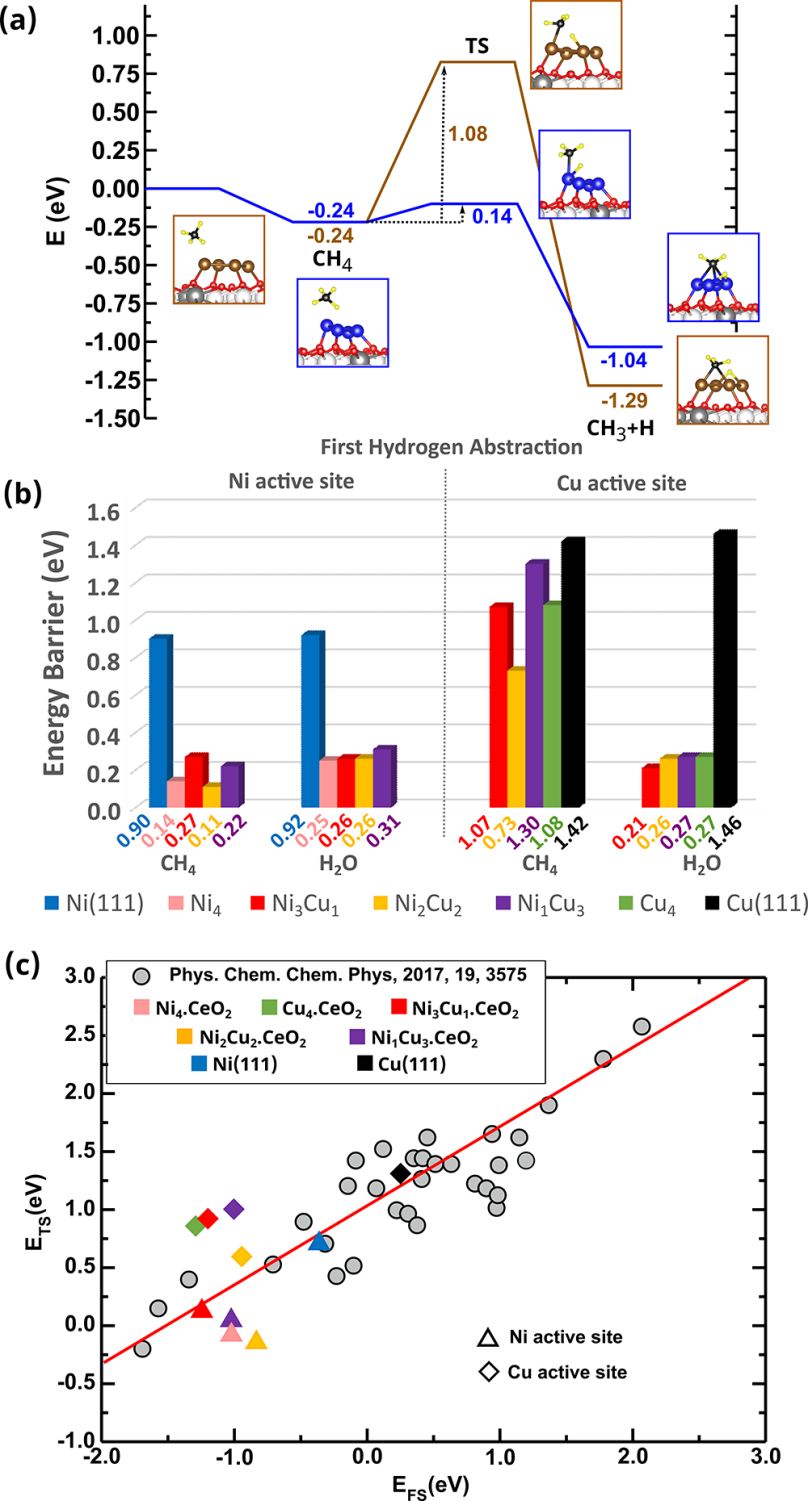
(a) Energy profile for the CH_4_ → CH_3_+H reaction on Ni_4–*x*_Cu_*x*_ (*x* = 0 to 4) clusters on CeO_2_(111). The structures shown on the left and right of the reaction pathways correspond to the side views of the optimized initial (molecularly adsorbed) and final (dissociated) states used in the search of the transition state structure (TS). (b) Lowest activation energy barriers for the CH_4_ → CH_3_+H and H_2_O → OH+H reactions channels offered by Ni and Cu sites in the Ni_4–*x*_Cu_*x*_ clusters (*x* = 0 to 4) on CeO_2_(111) and for the extended Ni and Cu(111) surfaces. (c) Scaling relation for the surface-stabilized pathway (*E*_TS_ = 0.67*E*_FS_ + 1.04, red line), according to ref (37). Shown are the *E*_TS_ and *E*_FS_ values for the paths with lowest activation barriers among those over Ni and Cu sites in the Ni_4–*x*_Cu_*x*_ clusters (*x* = 0 to 4) on CeO_2_(111) and for the extended Ni(111) and Cu(111) surfaces (not included in the linear fit).

In Figure 2a, the dissociation products correspond to stable chemisorbed states geometrically close to the corresponding transition state structures, with both CH_3_ and H species on the metal clusters. We observe that paths that involve simultaneous interactions of methane with the Ni or Cu sites and oxygen centers of the ceria support (Figure S4), with CH_3_ species adsorbed on the metal clusters and H forming OH species in the final states, did not lead to lower activation barriers when compared with the cases in which both CH_3_ and H species bind to the chemically modified supported cluster. This is similar to the case of Pt_4_ clusters on CeO_2_(111).^22^

The factors leading to the better ability of the Ni/CeO_2_ system to cleave the first C–H bond, as compared with Cu/CeO_2_, are related to the distinct adsorption properties of the former. The careful inspection of the adsorption of the CH_4_ molecule on these systems reveals that on Ni_4_.CeO_2_, the molecule comes closer to the surface than on Cu_4_.CeO_2_, cf. the C–Ni distance of 212 pm and C–Cu of 261 pm (Table S4, Figures S5a and S6a). The closer approach can be correlated with the clear difference between the *d* and *d*_*z*^2^_ projected density of states on the Ni and Cu atom over which CH_4_ dissociates, with approximately 67% and 99% occupation of the *d*_*z*^2^_ states, respectively (Figure S7, Table S2). Therefore, the Pauli repulsion to the methane’s frontier orbital is reduced in the case of the Ni_4_.CeO_2_ surface. Furthermore, the comparison with the corresponding *d*_*z*^2^_ occupancy of approximately 84% and 100% in the free-standing Ni_4_.gas and Cu_4_.gas clusters, respectively (Table S2), reveals that in the case of Ni_4_, the *d*_*z*^2^_ states become less occupied upon adsorption of the metal cluster onto the ceria surface, resulting in the partial depopulation of the *d*_*z*^2^_ states (from 84% to 67%) that facilitates the approach of the CH_4_ molecule and the easier cleavage of the first C–H bond. Such ligand effect is a consequence of the bonding of the metal atoms of the cluster to the surface oxygen atoms of the ceria support, which is much more pronounced in the case of small supported Ni clusters.

In both Ni_4_.CeO_2_ and Cu_4_.CeO_2_ systems, charge is transferred to the CH_4_ molecule upon adsorption, which is significantly larger for Ni_4_.CeO_2_ than for Cu_4_.CeO_2_, as reflected by the corresponding increase in the Bader charge of the C atom, namely, 0.16 |*e*^–^| and 0.06 |*e*^–^| for Ni_4_.CeO_2_ and Cu_4_.CeO_2_, respectively, with respect to the gas-phase molecule (Table S3). Most importantly, the C–H bond that will ultimately be cleaved is more elongated for Ni_4_.CeO_2_ than for Cu_4_.CeO_2_ (cf. 119 and 111 pm, respectively, Table S3, Figures S5a and S6a, with 110 pm in the gas-phase), facilitating bond breaking.

We will now address the effect of alloying Ni with Cu on the activation barrier for the CH_4_ to CH_3_+H reaction. For extended Cu-doped Ni surfaces, it has been found that barriers over Ni sites are larger for the Cu-doped surfaces than for the corresponding undoped ones.^38,39^ For example, for the case of the Ni(111) with a surface Cu dopant concentration of 1/4, the barrier is 1.3 times higher.^38^ The CH_4_ to CH_3_+H reaction channels with the lowest activation energy barriers offered by the Ni and the Cu sites of the CeO_2_(111)-supported bimetallic Ni_4–*x*_Cu_*x*_ clusters (*x* = 1 to 3) are exothermic with Δ*E* values within the −0.5 to −1.1 eV energy range (Table S3). Figure 2b compares the corresponding activation energy barriers (cf. Figure S4). It is evident that barriers are smaller for Ni sites, lying within the 0.1–0.3 eV energy interval, whereas those over Cu sites lie within a 0.7–1.3 eV interval. As in the case of the extended Cu-doped Ni surfaces reported in the literature,^38^ the lower energy barrier over Ni sites on the bimetallic Ni_4–*x*_Cu_*x*_.CeO_2_ surfaces could be up to a factor of 2 higher than over the corresponding Ni site on the monometallic Ni_4_.CeO_2_ surface due to the presence of the neighboring Cu atoms, although barriers are never higher than 0.3 eV (cf. for example 0.14 and 0.27 eV for Ni_4_.CeO_2_ and Ni_3_Cu_1_.CeO_2_, respectively, Figures 2b and S4). These changes can be attributed to the interacting electronic effects of Ni and Cu atoms. For example, comparing the Ni-projected density of states for the d-orbitals of the Ni atom over which CH_4_ dissociates over the Ni_4_.CeO_2_ and Ni_3_Cu_1_.CeO_2_ surfaces (cf. atom number 4 in Figure S7, Table S2), we observe that adding Cu results in the lowering of the Ni *d*-band center and the partial population of the *d*_*z*^2^_ orbitals (from 67% to 79%), which leads to a decrease in molecule–surface interaction for Ni_3_Cu_1_.CeO_2_, as reflected by a CH_4_ molecule positioned further away from the surface (from 212 for Ni_4_.CeO_2_ to 270 pm Ni_3_Cu_1_.CeO_2_, Table S4, Figures S5a and S8a), and a less charge transferred to the alkane molecule upon adsorption (from 0.16|*e*^–^| for Ni_4_.CeO_2_ to 0.08|*e*^–^| for Ni_3_Cu_1_.CeO_2_, Table S4). Furthermore, since Ni sites proved to be highly reactive for abstracting H from chemisorbed CH_4_ molecules, the reactivity differences have been correlated to the binding of the H atom to the supported clusters (with respect to 1/2 H_2_), calculated by removing the CH_3_ species from the TS structures, without further optimization (Table S5, Figure S16).

In the case of the Cu sites on the Ni_4–*x*_Cu_*x*_.CeO_2_ surfaces, energy barriers are higher than 0.7 eV, and somewhat more pronounced variations are observed with the composition of the cluster than those found for Ni sites (Figure 2b), with the lowest barrier of 0.73 eV for the Ni_2_Cu_2_ cluster. Inspection of the Cu-projected densities of states (Figure S7, Table S4) does not reflect noticeable differences in the population of the d_z^2^_ orbitals (∼99%) of Cu sites in the Ni_4–*x*_Cu_*x*_.CeO_2_ (*x* = 1–4) surfaces. However, differences in the activation barriers on Cu sites correlate with the binding of the H atom to the supported clusters (with respect to 1/2 H_2_), calculated by removing the CH_3_ species from the TS structures, without further optimization, according to which the stronger the H binding, the more stabilized the TS structure will be, and the smaller the barrier. The binding of the H atom on the Ni_2_Cu_2_ cluster is approximately 0.2 eV stronger, as compared with Ni_3_Cu_1_ and Ni_1_Cu_3_ (Table S5, Figure S17). Furthermore, inspection of the transition state structures for activation on Cu sites (Table S6, Figures S6b, S8h, S9e, S10e) reveals geometrical differences, particularly with regard to the H atom in the generally elongated C–H bond. The higher stabilization of the TS structure for the CH_4_ to CH_3_+H reaction over Cu sites in the Ni_2_Cu_2_ cluster can be related to the fact the H atom is close to two Ni atoms, with Ni–H distances of 187 and 194 pm (Table S6), compared with one Ni atom in the Ni_3_Cu_1_ and Ni_1_Cu_3_ clusters, with Ni–H distances of 190 and 213 pm, respectively. We further note that the adsorption energy of a H atom after geometry optimization on the supported clusters follows the the order of Ni_4_ > Ni_2_Cu_2_ > Ni_3_Cu_1_ ∼ Ni_1_Cu_3_ > Cu_4_ (Figure S17).

Summarizing, as stated above, the presence of Ni sites in bimetallic Ni_4–*x*_Cu_*x*_ clusters on CeO_2_(111) will always be the reason why these clusters will be highly active for the CH_4_ to CH_3_+H reaction because the energy barriers are less than 0.3 eV. The activity of Ni and inactivity of Cu in the bimetallic Ni_4–*x*_Cu_*x*_ clusters is controlled by ligand effects, which are much more pronounced in the case of Ni sites than on Cu ones.

As previously discussed for the case of the ceria-supported monometallic Ni_4_ cluster,^22^ strong interactions between ceria and the small clusters lead to the stabilization of the CH_3_+H products, as well as the CH_4_ molecule in ways that the cleavage of the first C–H bond is significantly easier than predicted by a simple scaling relation^37^ between the calculated energies of the transition state structures for methane activation, *E*_TS_ (referenced to gas-phase CH_4_ and the clean surface) and the energy of the CH_3_+H final state, *E*_FS_. Similarly, for the reaction channels offered by Ni sites in the Ni_4–*x*_Cu_*x*_ clusters (*x* = 0 to 3) on CeO_2_, substantial deviations of up to 0.7 eV from predicted *E*_TS_ values are observed (Figure 2c, Table S3), implying calculated activation barriers up to 0.7 eV lower than predicted. However, the contrary is observed for the Cu sites (Figure 2c, Table S3), with *E*_TS_ values that are up to 0.7 eV higher than the ones predicted by the linear scaling relation. Despite the relatively high stability of the final state of the reaction channels offered by the Cu sites, the nearly fully populated Cu *d* and *d*_*z*^2^_ states (Table S2) do not allow initial adsorption states that facilitate the breaking of the C–H bond, and consequently, the activation barriers are high (Figure 2d). These results illustrate that the *nature* of the metal site in low-loaded bimetallic-oxide systems and strong metal–support interactions are crucial to obtaining an improved activity toward the first C–H bond activation. However, what happens in the case of the decomposition of the methyl CH_3_ species when alloying Ni with Cu?

As mentioned above, in the case of low-loaded monometallic Ni/CeO_2_ catalysts, not only the cleavage of the first C–H bond from CH_4_ is easy but also the full decomposition of the formed CH_3_ species.^12,15,17,20^ The calculated reaction profile for the dissociation from CH_4_ up to CH over the Ni_4_.CeO_2_ model catalyst is shown in Figure 3. The first H abstraction produces CH_3_ and H species bound to the Ni cluster with a very low activation barrier of 0.14 eV, as discussed above. The subsequent dissociation of the adsorbed CH_3_ produces directly CH and 2H species, also bound to the Ni cluster, with a barrier of 0.63 eV. The reaction CH_4_ → CH_3_+H → CH + 3H is exothermic with Δ*E*= −0.6 eV, and the highest barrier of 0.63 eV can be overcome under typical experimental conditions for the dry or steam reforming of methane,^12,17^ as well as those for its conversion to methanol.^15^ Further dissociation of the CH species produces C adatoms.^12,17^ In the case of the direct oxidation of methane to methanol, making the full decomposition of CH_3_ species energetically unfavorable is crucial for improving reaction selectivity, that is, hindering the path that would lead to CO/CO_2_ formation.

**Figure 3:**
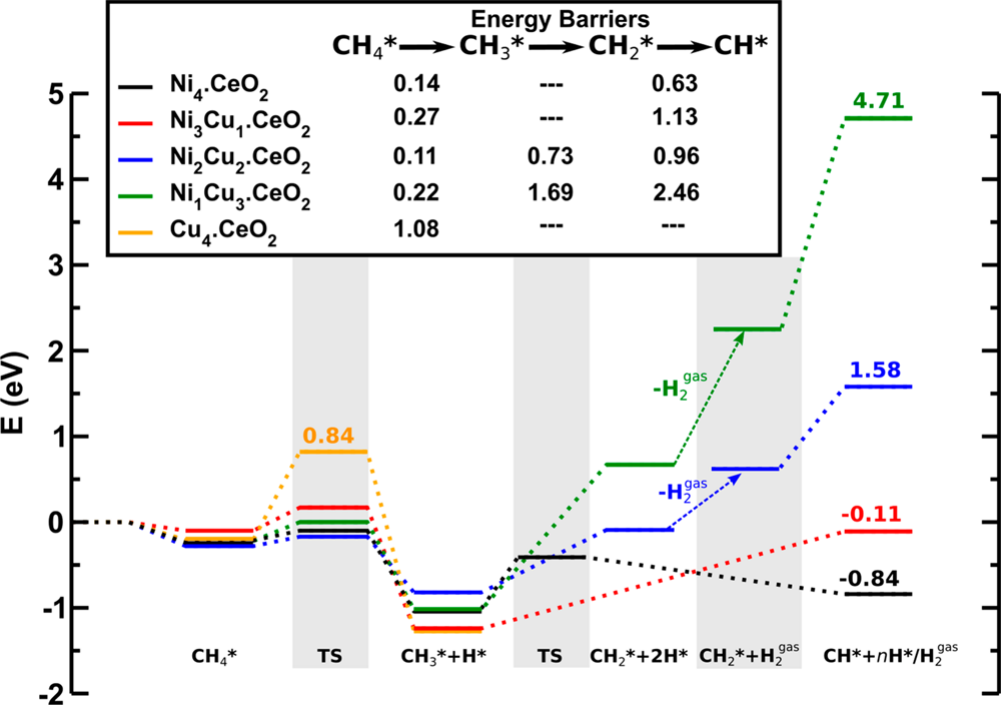
Energy profile for the CH_4_ decomposition to CH on Ni_4–*x*_Cu_*x*_ (*x* = 0 to 4) clusters on CeO_2_(111). Energies of all states are referred to those of the clean surface and gas-phase CH_4_. The stars indicate chemisorbed species, and the arrows represent the removal of chemisorbed hydrogen as gas-phase H_2_.

The dramatic synergistic effect of ceria-supported bimetallic Ni_4–*x*_Cu_*x*_ clusters (*x* = 1 to 3) on the decomposition of methyl species is shown in Figure 3. As discussed above, the first H abstraction will always occur over Ni sites. However, the combination of Ni with a small amount of Cu, forming the Ni_3_Cu_1_.CeO_2_ surface, induces important changes in the energy profile from CH_4_ to CH, as compared with that for the monometallic Ni_4_.CeO_2_ system. The CH_4_ → CH_3_+H → CH + 3H reaction over the bimetallic Ni_3_Cu_1_.CeO_2_ surface is slightly exothermic with a minimal energy gain (thermoneutral). The lowest activation barrier for the cleavage of the first C–H bond from CH_4_ over Ni sites amounts to 0.27 eV as discussed above (Figure 2b), but the following dissociation of CH_3_+H that produces CH+3H species, has a barrier of at least 1.13 eV (Figure 3). The potential energy surface goes uphill and no transition state structure has been found. The barrier for the decomposition of the CH_3_ species over the bimetallic Ni_3_Cu_1_.CeO_2_ surface is approximately 1.8 times higher than that for the monometallic Ni_4_.CeO_2_ surface with a barrier of 0.63 eV, as discussed above.

The higher activation barrier for the CH_3_+H → CH + 3H reaction over the bimetallic Ni_3_Cu_1_.CeO_2_ surface, compared with Ni_4_.CeO_2_, can be related to the significantly lower stability of the CH + 3H final state in Ni_3_Cu_1_.CeO_2_ (cf. – 0.11 and −0.84 eV for Ni_3_Cu_1_.CeO_2_ and Ni_4._Cu_1_.CeO_2_, respectively). We observe that the final state is to a great extent stabilized by the binding of the H species. The binding of the 3H atoms to the supported clusters (with respect to 1/2 H_2_), calculated by removing the CH species from the final state structures, without further relaxation, is −0.13 and −1.15 eV for Ni_3_Cu_1_.CeO_2_ and Ni_4_.CeO_2_, respectively. On the Ni_3_Cu_1_.CeO_2_ surface, two H atoms bind to one Ni atom (Ni–H: 146 and 157 pm) and the other H forms a bridge between Ni and Cu atoms (Ni–H: 176 pm and Cu–H: 166 pm), whereas on Ni_4_.CeO_2_, two H atoms bind in bridge position between two Ni atoms (2× Ni–H: 159 and 160 pm) and the other binds to a single Ni atom (Ni–H: 157) (Figure S18).

Finally, we briefly mention the effect of adding more Cu (50 to 75%) to the bimetallic Ni_1–*x*_Cu_*x*_.CeO_2_ model catalyst on the decomposition of methyl species. In contrast to the Ni_4_.CeO_2_ and Ni_3_Cu_1_.CeO_2_ surfaces for which CH+2H species are produced, over the Ni_2_Cu_2_.CeO_2_ and Ni_1_Cu_3_.CeO_2_ surfaces, the dissociation of CH_3_ species initially produces CH_2_+H (Figure 3). In both cases, the reaction is highly endothermic with barriers of at least 0.73 and 1.69 eV for the Ni_2_Cu_2_.CeO_2_ and Ni_1_Cu_3_.CeO_2_ surfaces, respectively. Accordingly, as for the bimetallic Ni_3_Cu_1_.CeO_2_ system discussed above with a very low proportion of Cu, the decomposition of CH_3_ species would also be suppressed if the proportion of Cu further increases.

The above results suggest that the full decomposition of CH_3_ species to C observed for low-loaded Ni/CeO_2_ systems,^12,15,17,20^ could already be avoided by mixing small amounts of Cu with Ni. This implies that the selectivity of the conversion of CH_*x*_ groups into high value chemicals such as methanol or other hydrocarbon species can be controlled by the so-called geometric ensemble effects in catalysis.^40,41^ In practical terms, by adding small amounts of an inactive metal such as Cu to form a bimetallic Ni_1–*x*_Cu_*x*_/CeO_2_ catalyst, the large ensembles of Ni sites that lead to full decomposition of CH_3_ and deactivation by carbon deposition would not exist, which corresponds to a geometric or structural effect.

In brief, for low-loaded Ni/CeO_2_ catalysts, it is the high reactivity of chemisorbed CH_4_ toward decomposition over small Ni particles, chemically modified by the presence of the reducible ceria support, that activates the formation of CH_3_ species and the subsequent dehydrogenation reactions. The full CH_4_ decomposition reaction pathway can be modified by employing ceria-supported bimetallic Ni_1–*x*_Cu_*x*_ particles. Through tuning the Ni/Cu atomic ratios in the bimetallic Ni_*x*_Cu_*y*_ particles, the extent of the Ni ensembles can be controlled, separating them by inert Cu for C–H bond cleavage, which leads to the suppression of the dehydrogenation of the CH_3_ species. To verify this hypothesis, we have calculated the complete reaction pathway for the direct oxidation of methane to methanol with oxygen: CH_4_ + O → CH_3_ + H + O → CH_3_O + H → CH_3_OH for the example of Ni_3_Cu_1_.CeO_2_, compared with Ni_4_.CeO_2_. In line with our previous work,^15^ an O atom is preadsorbed on both metallic clusters (differences between the values reported in this and previous work for Ni_4_.CeO_2_,^15^ are related to the inclusion vdW interactions in this work). The results indicate that the first hydrogen abstraction from CH_4_, in the presence of O species, has relatively low barriers, between 0.4 and 0.6 eV for both surfaces (Figure 4). The cleavage of a C–H bond from the CH_3_ species is more likely to occur on Ni_4_.CeO_2_, since it has an activation barrier which is by 0.38 eV lower than that for formation of methoxy species (Figure 4). However, on Ni_3_Cu_1_.CeO_2_, the formation of methoxy species becomes probable since the activation energy for the formation of CH_3_O species is comparable—or even lower—than that for the further dehydrogenation of CH_3_ species (Figure 4). We further calculated the formation of CH_3_OH from CH_3_O+H species and found that it is favored over the Ni_3_Cu_1_.CeO_2_ surface compared with Ni_4_.CeO_2_, since the activation barrier is by 0.23 eV lower for the supported Ni_3_Cu_1_ cluster. Hence, the addition of Cu to ceria-supported Ni particles could increase the selectivity toward methanol formation from CH_4_ and O_2_.

**Figure 4:**
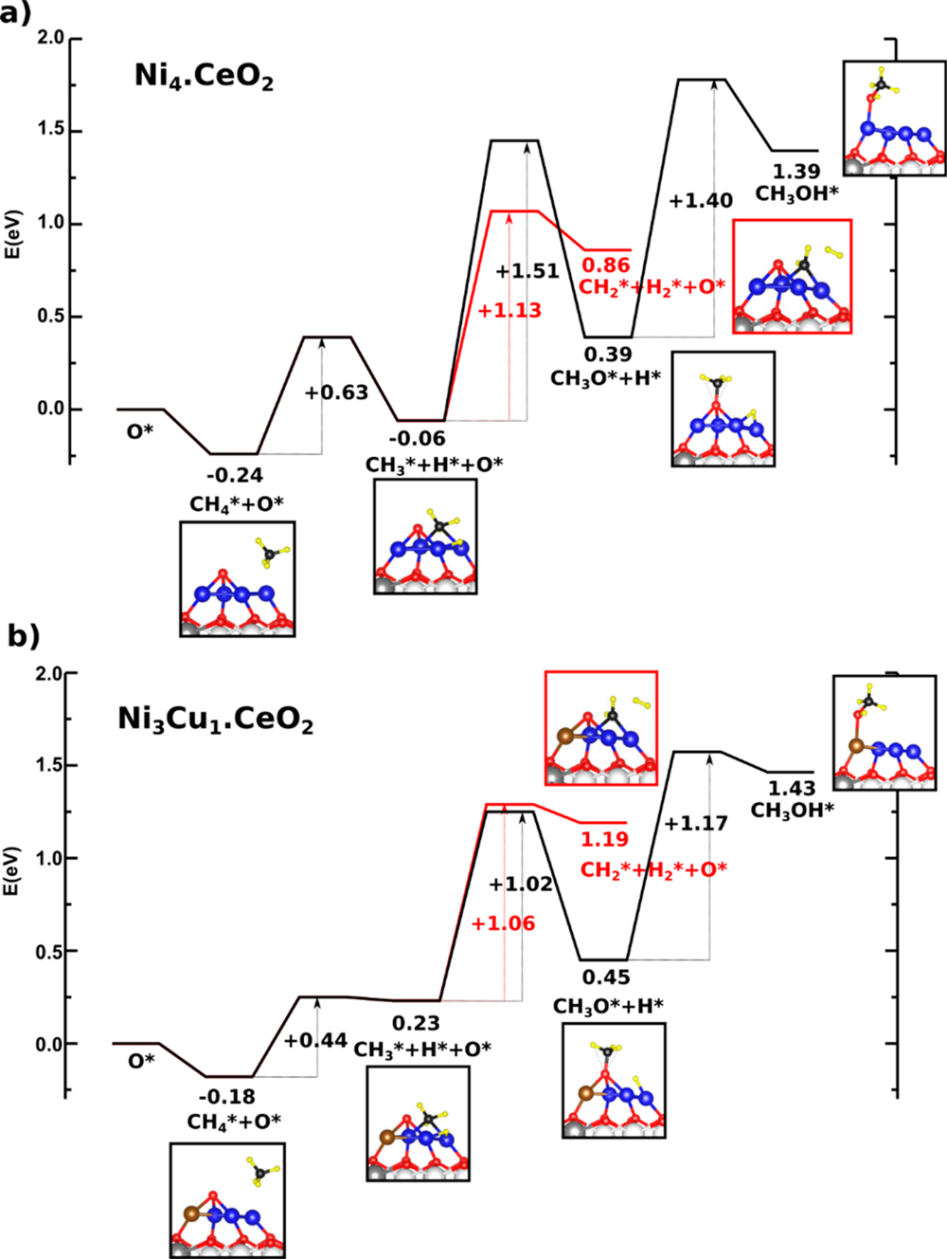
Full reaction mechanism for the direct conversion of methane to methanol from chemisorbed CH_4_ and O species over (a) Ni_4_.CeO_2_ and (b) Ni_3_Cu_1_.CeO_2_ surfaces.

In the following, we briefly address the role of active site isolation in the reactivity of Ni_1–*x*_Cu_*x*_/CeO_2_ systems for O–H bond cleavage from water, because water was found to enable the catalytic conversion of CH_4_ to methanol over monometallic Ni/CeO_2_ surfaces by site-blocking.^15^

The dissociation of H_2_O on the extended Ni(111) and Cu(111) surfaces at low temperatures is very difficult because of large energy barriers of 0.92 and 1.46 eV, respectively (cf. Figures 2b and S2), in line with previous results.^19,42^ Earlier experimental and theoretical studies have shown that on low-loaded Ni/CeO_2_ systems,^12,15,19^ water binds strongly and would easily dissociate at interfacial Ni sites (Ni^δ+^) in direct contact with the CeO_2_ support, where Ni and O sites can work cooperatively, that is, in the final state, OH species are bound to the Ni nanoparticle, whereas H species form OH species with surface oxygen atoms. Over the Ni_4_.CeO_2_ model catalyst, the barrier for such a cooperative reaction pathway is 0.25 eV (Figure 2b and S4), whereas those for noncooperative pathways over Ni^δ+^ sites are within the 0.4–0.5 eV range (Figures S4). Similarly, on all of the Ni_4–*x*_Cu_*x*_.CeO_2_ surfaces (*x* = 1 to 3), the strong binding of water molecules on Ni sites (Figures S19–S22) and their easy dissociation are predicted, with activation barriers within the 0.2–0.3 eV range for cooperative pathways (Figures 2b and S4) and the 0.3–0.7 eV range for noncooperative ones (Figure S4).

As for the interfacial Cu sites (Cu^δ+^), contrary to the extended metallic Cu(111) surface that does not show reactivity toward H_2_O dissociation, but similarly to Ni sites (Ni^δ+^) in the Ni_4–*x*_Cu_*x*_.CeO_2_ systems, water binds strongly and would dissociate with or without participation of the ceria support with barriers that are below 0.3 eV (Figures 2b and S4). Therefore, it can be concluded that Ni sites in low-loaded bimetallic Ni_4–*x*_Cu_*x*_ clusters (*x* = 0 to 3) on CeO_2_(111) will bind and easily activate both CH_4_ and H_2_O, whereas Cu sites will be active toward H_2_O dissociation but *not* CH_4_. The reason for the distinct behavior of the Cu sites can be related to the higher initial stabilization of molecular water as compared to methane on such sites. For example, the initial adsorption energy of CH_4_ and H_2_O molecules on the monometallic Cu_4_.CeO_2_ surface is −0.24 and −0.81 eV, respectively (Figures S12 and S23, Tables S4 and S8), with Cu–C and Cu–O bond distances of 261 and 194 pm, respectively. Moreover, the O–H bond in the molecularly adsorbed water that will eventually break is somewhat more elongated than the other (cf. 101 and 98 pm, Table S8), while the corresponding difference between the C–H bonds is smaller (cf. 111 and 110 pm, Table S4).

In summary, this work has addressed the difficult problem of designing a catalyst that would allow the control of the degree of dehydrogenation of CH_3_ species that form after the cleavage of the first C–H bond in CH_4_ and at the same time is active for the possibly simultaneous dissociation of H_2_O. NiCu bimetallic clusters supported on the CeO_2_(111) surface with different Ni/Cu ratios were investigated as model catalysts for the activation of C–H and O–H bonds. On the basis of the results obtained in this work, as well as on the previous knowledge on systems consisting of monometallic Ni clusters supported on ceria, it can be concluded that the presence of Ni sites in the NiCu bimetallic clusters is essential for C–H bond breaking in CH_4_, where strong interactions between the small metal clusters in direct contact the with the ceria support lead to the stabilization of both the CH_4_ molecule and the CH_3_+H dissociation product, producing active and stable catalysts for methane activation under very mild conditions. Moreover, CH_3_ species fully decompose on Ni ensembles. A design strategy is here suggested to tune the degree of dehydrogenation of CH_3_ species that consists of mixing Ni with Cu such that the surface Ni ensembles in the NiCu bimetallic particles only allow for the cleavage of the desired number of C–H bonds in CH_3_. By using such catalysts, the high reactivity toward breaking the first C–H bond in the alkane will not be affected, but the outcome of its conversion will, because CH_3_ species will not decompose on Cu. The results also show that the strategy does not affect the ability of Ni/CeO_2_ catalyst for O–H bond cleavage. The findings underscore the idea that by tuning the composition of supported bimetallic nanoparticles as well as manipulating metal–support interactions, various selectivities can be obtained.

## Supporting Information Available

Detailed description of the computational methods and models. Additional data on the electronic structure and Bader charges. All reaction pathways with initial, transition and final state structures considered in this work, as well as the binding of a H atom, as reactivity probe (PDF)

## Notes

The DFT data that support the findings of this study are available in Materials Cloud {https://www.materialscloud.org/home} with the identifier DOI: 10.24435/materialscloud:8y-7m. The data is also available from the authors upon reasonable request.
