# Supplementary material for: Tuning Selectivity in the Direct Conversion of Methane to Methanol: Bimetallic Synergistic Effects on the Cleavage of C–H and O–H Bonds over NiCu/CeO2 Catalysts
Source: J Phys Chem Lett. 2022 Jun 14;13(24):5589–96. doi: 10.1021/acs.jpclett.2c00885 (PMC9234976; doi:10.1021/acs.jpclett.2c00885)
Supplement: Supplementary file 1 — jz2c00885_si_001.pdf [file jz2c00885_si_001.pdf]

Supporting Information

Tuning Selectivity in the Direct Conversion of Methane to Methanol: Bimetallic Synergistic Effects on the Cleavage of C–H and O–H Bonds over NiCu/CeO_2 Catalysts

Pablo G. Lustemberg,[*,†,‡] Sanjaya D. Senanayake,[¶] José A. Rodriguez ,[¶] and M. Verónica Ganduglia-Pirovano[†]

†*Instituto de Catálisis y Petroleoquímica, CSIC, C/Marie Curie 2, 28049 Madrid, Spain*

‡*Instituto de Física Rosario (IFIR), CONICET-UNR, Bv 27 de Febrero 210bis, S2000EZP Rosario, Santa Fe, Argentina*

¶*Chemistry Division, Brookhaven National Laboratory, Upton, New York 11973, United States*

E-mail: p.lustemberg@csic.es

Theoretical Models and Computational Methods

All electronic structure calculations were carried out using the spin-polarized DFT approach, as implemented in the Vienna ab initio simulation package (VASP) (vasp site, http://www.vasp.at; version vasp.5.3.5).[1,2] Ce (4f, 5s, 5p, 5d, 6s), O (2s, 2p), Ni (3p, 3d, 4s) and Cu (3p, 3d, 4s) electrons were explicitly treated as valence states within the projector

augmented wave (PAW)[3] method with a plane-wave cutoff energy of 415 eV, whereas the remaining electrons were considered as part of the atomic cores. Total energies and forces were calculated with a precision of 10^{-6} eV and 10^{-2} eV/Å for electronic and force convergence, respectively, within the DFT+U approach by Dudarev et al.[4] (Ueff = U – J = 4.5 eV for the Ce 4f electrons) with the generalized gradient approximation (GGA) proposed by Perdew, Burke, and Ernzerhof (PBE),[5] including long-range dispersion corrections, employing the so-called DFT-D3 approach.[6,7]

The $Ni_{4-x}Cu_x.CeO_2(111)$ (x = 1,.,4) model catalysts consist of a flat four-atom bimetallic cluster on the $CeO_2(111)$ surface. They were modelled employing surface unit cells with 3 × 3 periodicity, with the calculated ceria bulk equilibrium lattice constant (CeO_2: 5.485 Å), and a two CeO_2-tri-layer slab. In all surface models, consecutive slabs were separated by at least a 12 Å-thick vacuum layer to avoid interaction between the slabs and their periodic images. Monkhorst-Pack grids with a 2 × 2 × 1 k-point sampling were used. All metal/ceria models used in this work are shown in Figure 1 in the main text. All atoms in the bottom O–Ce–O tri-layer were kept fixed at their optimized bulk-truncated positions during geometry optimization, whereas the rest of the atoms were allowed to fully relax.

The adsorption energies of methane and water were calculated according to the following equation for the example of the dissociative adsorption of methane:

$$E_{ads} = E[(\mathrm{CH_3} + \mathrm{H})/\mathrm{Ni}_{4-x}\mathrm{Cu}_x.\mathrm{CeO_2}(111)] - E[\mathrm{Ni}_{4-x}\mathrm{Cu}_x.\mathrm{CeO_2}(111)] - E[\mathrm{CH}_{4gas}] \quad (1)$$

where $E[(CH_3+H)/Ni_{4-x}Cu_x.CeO_2(111)]$ is the total energy of the methyl and hydrogen species co-adsorbed on the surface, $E[Ni_{4-x}Cu_x.CeO_2(111)]$ is the total energy of the surface without the adsorbate, $E[CH_{4gas}]$ is the energy of the methane molecule in the gas phase. No ZPE corrections have been considered.

To locate transition state (TS) structures, we employed the climbing image nudged elastic band method (CI-NEB)[8,9] with nine images for each reaction pathway. For all the TS

reported in this work, we have found only one imaginary frequency, and the full geometry optimizations starting from its back and forward nearest configurations (along the reaction path) ended in a non-dissociated and dissociated state, respectively.

In the calculated potential energy profiles, the energy barrier, $E_{Barrier} = E_{TS} - E_{IS}$, equals the difference between the energy of the transition state, E_{TS}, and the initial (molecularly chemisorbed) state, E_{IS}, whereas the effective or apparent energy barrier is given by the energy of the transition state, E_{TS}, referenced to gas-phase CH_4 and the clean surface.

Models Stability

The results in Figure S1 indicate that as the size of the clusters increases, the stability per atom increases, in line with previous work.[10] The supported flat Ni_4, Cu_4 and Ni_3Cu_1 clusters are slightly less stable than the corresponding pyramidal ones, for which only the atoms in direct contact with the support are oxidized. In addition, the formation energies of the flat Ni_4 (Ni_4.flat), pyramidal (Ni_4.pyr), as well as the flat and pyramidal Ni_3Cu_1 clusters (Ni_3Cu_1.flat and Ni_3Cu_1.pyr) were calculated with respect to the (flat) Ni_3 cluster and an adsorbed isolated Ni_1/Cu_1 atom, according to the following equations:

$E(Ni_4.flat) + E(CeO_2) - E(Ni_3) - E(Ni_1) = -0.27$ eV

$E(Ni_4.pyr) + E(CeO_2) - E(Ni_3) - E(Ni_1) = -0.39$ eV

$E(Ni_3Cu_1.flat) + E(CeO_2) - E(Ni_3) - E(Cu_1) = -0.45$ eV

$E(Ni_3Cu_1.pyr) + E(CeO_2) - E(Ni_3) - E(Cu_1) = -0.73$ eV

In all cases, the formation energy is negative, which indicates that the formation of these structures are energetically favored. Although this information is relevant, it would be incomplete without the calculation of diffusion barriers of adsorbed Cu_1 species to attach to Ni_3 clusters, forming Ni_3Cu_1 flat clusters, followed by the pathway for the formation of Ni_3Cu_1.pyr from Ni_3Cu_1.flat (see Figure S1). The results in Figure S1 reveal that the diffusion of an isolated Cu_1 species and attachment to the Ni_3 cluster to form the Ni_3Cu_1.flat cluster is likely to occur since the barrier is as low as 0.33 eV, however, the formation of a pyramidal

Ni_3Cu_1.pyr structure (Ni_3Cu_1.pyr.1) from the Ni_3Cu_1.flat has a barrier of 0.85 eV, which is 2.57 times higher. In short, kinetic considerations also support the choice of the model systems in this study.

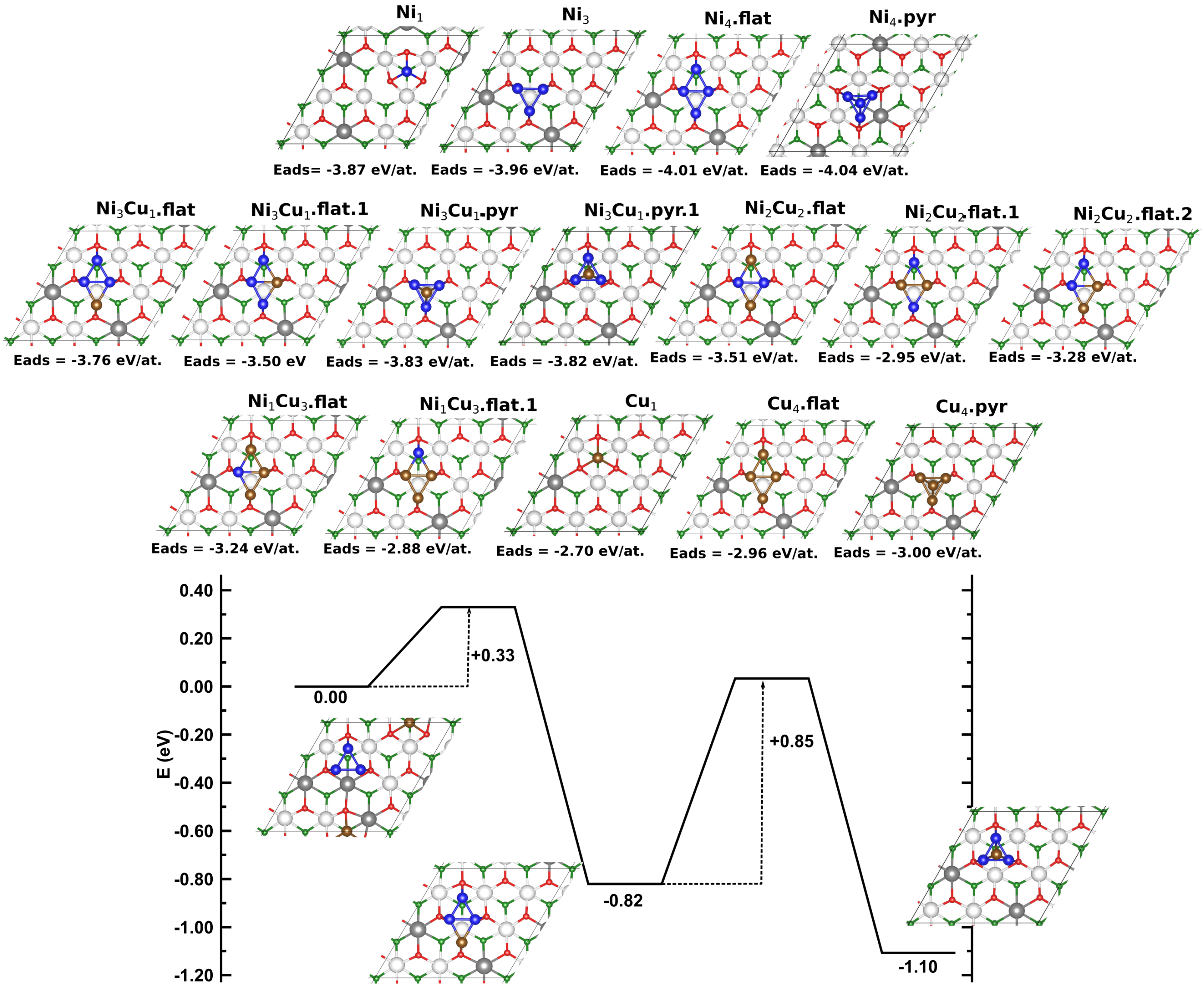

Figure S1: Adsorbed $Ni_{4-x}Cu_x$ (x=0 to 4) on $CeO_2(111)$. The average adsorption energy of $Ni_{4-x}Cu_x$ species is listed below each structure in eV per metallic atom. The pathway for the formation of the Ni_3Cu_1.flat cluster from the adsorbed Ni_3 cluster and the diffusion of Cu_1 species, followed by the pathway for the formation of a Ni_3Cu_1.pyr from the Ni_3Cu_1.flat is shown. Ni and Cu atoms are depicted in blue and brown, respectively, while surface/subsurface oxygen atoms are in red/green, Ce^{4+} in white, and Ce^{3+} in gray.

Table S1: Bader charges, computed as differences with respect to the nominal charges of the corresponding isolated atoms. (a) Supported clusters $Ni_{4-x}Cu_x.CeO_2$. (b) Bimetallic nanoparticle in the gas phase ($Ni_{4-x}Cu_x$.gas), i.e., the free-standing $Ni_{4-x}Cu_x$ clusters, which result from the removal of the $CeO_2(111)$ support from the $Ni_{4-x}Cu_x.CeO_2$ systems, without further optimization of the geometry. Each atom is labeled following the numbering in Figure 1 in the main text. Numbers in blue (brown) refer to the Ni (Cu) species.

(a) Δq $\mathbf{Ni_{4-x}Cu_x.CeO_2}$					
**Catalyst**	**at. 1**	**at. 2**	**at. 3**	**at. 4**	**Total**
$Ni_4.CeO_2$	−0.360	−0.217	−0.281	−0.232	−1.090
$Ni_3Cu_1.CeO_2$	−0.332	−0.237	−0.263	−0.242	−1.075
$Ni_2Cu_2.CeO_2$	−0.290	−0.264	−0.260	−0.251	−1.066
$Ni_1Cu_3.CeO_2$	−0.323	−0.237	−0.263	−0.261	−1.083
$Cu_4.CeO_2$	−0.343	−0.233	−0.280	−0.224	−1.080
(b) Δq $\mathbf{Ni_{4-x}Cu_x.gas}$					
**Catalyst**	**at. 1**	**at. 2**	**at. 3**	**at. 4**	**Total**
$Ni_4.CeO_2$	0.117	−0.116	0.112	−0.113	0.000
$Ni_3Cu_1.CeO_2$	0.087	−0.150	0.125	−0.150	−0.088
$Ni_2Cu_2.CeO_2$	0.134	−0.184	0.134	−0.172	−0.088
$Ni_1Cu_3.CeO_2$	0.106	−0.140	0.100	−0.152	−0.087
$Cu_4.CeO_2$	0.119	−0.163	0.115	−0.158	−0.086

Table S2: Occupation percentage of the d and the d_{z^2} bands for the indicated atom position of each $Ni_{4-x}Cu_x.CeO_2$ model catalyst and free standing $Ni_{4-x}Cu_x$.gas clusters. d-band center values of $Ni_{4-x}Cu_x.CeO_2$ are also indicated.

$Ni_{4-x}Cu_x.CeO_2$				
(a) d band Occupation (%)				
**Catalyst**	**at. 1**	**at. 2**	**at. 3**	**at. 4**
$Ni_4.CeO_2$	71.75	72.74	73.07	72.72
$Ni_3Cu_1.CeO_2$	89.83	90.61	98.53	90.32
$Ni_2Cu_2.CeO_2$	98.60	91.04	98.64	91.03
$Ni_1Cu_3.CeO_2$	98.63	98.63	98.45	91.08
$Cu_4.CeO_2$	98.68	98.81	98.17	98.47
(b) d_{z^2} band Occupation (%)				
**Catalyst**	**at. 1**	**at. 2**	**at. 3**	**at. 4**
$Ni_4.CeO_2$	71.28	70.36	68.54	66.76
$Ni_3Cu_1.CeO_2$	88.75	81.72	99.13	79.42
$Ni_2Cu_2.CeO_2$	99.03	74.56	99.01	74.43
$Ni_1Cu_3.CeO_2$	99.26	99.38	99.07	71.04
$Cu_4.CeO_2$	99.40	99.28	98.59	99.15
(c) d band Center (eV)				
**Catalyst**	**at. 1**	**at. 2**	**at. 3**	**at. 4**
$Ni_4.CeO_2$	−1.25	−1.41	−1.27	−1.46
$Ni_3Cu_1.CeO_2$	−1.53	−1.75	−2.04	−1.78
$Ni_2Cu_2.CeO_2$	−1.89	−1.59	−1.99	−1.56
$Ni_1Cu_3.CeO_2$	−1.89	−2.09	−2.03	−1.55
$Cu_4.CeO_2$	−1.95	−2.22	−2.14	−2.21
$Ni_{4-x}Cu_x$.gas				
(d) d band Occupation (%)				
**Catalyst**	**at. 1**	**at. 2**	**at. 3**	**at. 4**
$Ni_4.CeO_2$	76.10	76.00	76.06	76.012
$Ni_3Cu_1.CeO_2$	87.47	88.41	97.61	88.38
$Ni_2Cu_2.CeO_2$	97.30	88.49	97.31	88.46
$Ni_1Cu_3.CeO_2$	97.77	98.20	97.76	88.53
$Cu_4.CeO_2$	97.86	98.22	97.83	98.17
(e) d_{z^2} band Occupation (%)				
**Catalyst**	**at. 1**	**at. 2**	**at. 3**	**at. 4**
$Ni_4.CeO_2$	85.10	84.02	85.29	83.97
$Ni_3Cu_1.CeO_2$	95.75	98.13	99.43	97.91
$Ni_2Cu_2.CeO_2$	99.44	95.93	99.46	95.87
$Ni_1Cu_3.CeO_2$	99.13	99.19	99.17	90.55
$Cu_4.CeO_2$	99.38	99.29	99.40	99.29

Table S3: Calculated energies (in eV) for the initial, E_{IS}, final, E_{FS}, and transition state structures, E_{TS}, for the $CH_4 \rightarrow CH_3 + H$ reaction over the $Ni_{4-x}Cu_x.CeO_2(111)$ as well as the Ni(111) and Cu(111) surfaces. All energies are relative to CH_4 in the gas phase and the corresponding clean surfaces. The predicted E_{TS} values correspond to the values obtained using the $E_{TS} = (0.67\ E_{FS} + 1.04)$ linear scaling relation for the actual calculated final state, E_{FS}. The predicted $E_{Barrier}$ values correspond to the activation energy barrier calculated as the energy difference between the predicted energy of the transition state and the calculated energy of the initial state. The model catalysts whose E_{TS} energy is less than zero is related to the fact that on them CH_4 binds relatively strongly, so that if the barrier for the first H abstraction from the chemisorbed CH_4 molecule is sufficiently low, E_{TS} will be negative when referenced to gas-phase CH_4 and the clean surface.

CH_4 First H abstraction									
**Catalyst**	**Reaction Channel**	$\mathbf{E}_{IS}$	$\mathbf{E}_{FS}$	$\mathbf{E}_{React}$	$\mathbf{E}_{TS}$ Pred.	$\mathbf{E}_{TS}$ Calc.	$\mathbf{E}_{Barrier}$ Pred.	$\mathbf{E}_{Barrier}$ Calc.	$\Delta\mathbf{E}_{TS} = \Delta\mathbf{E}_{Barrier}$
Ni(111)	Ni	−0.26	−0.35	−0.09	0.81	0.64	1.07	0.90	−0.17
Ni_4	Ni	−0.24	−1.04	−0.80	0.34	−0.10	0.58	0.14	−0.44
Ni_3Cu_1	Ni	−0.10	−1.24	−1.07	0.26	0.17	0.36	0.27	−0.09
	Cu	−0.26	−1.19	−0.93	0.24	0.81	0.50	1.07	+0.57
Ni_2Cu_2	Ni	−0.28	−0.82	−0.54	0.49	−0.17	0.77	0.11	−0.66
	Cu	−0.23	−0.97	−0.74	0.39	0.50	0.62	0.73	+0.11
Ni_1Cu_3	Ni	−0.22	−1.02	−0.80	0.36	0.00	0.58	0.22	−0.36
	Cu	−0.32	−1.00	−0.68	0.37	0.98	0.69	1.30	+0.54
Cu_4	Cu	−0.24	−1.29	−1.05	0.18	0.84	0.42	1.08	+0.66
Cu(111)	Cu	−0.26	+0.38	+0.64	1.29	1.16	1.55	1.42	−0.13

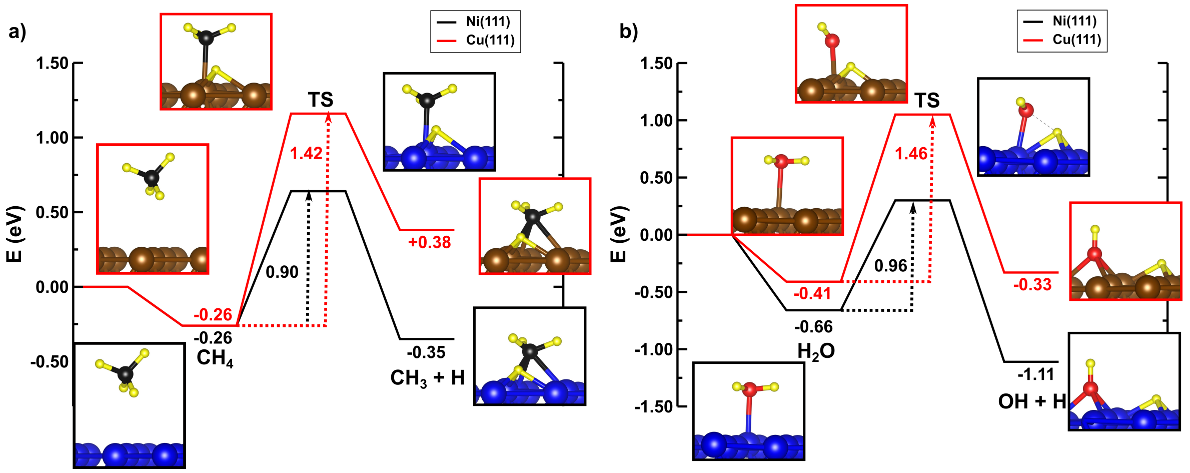

Figure S2: Reaction pathways for a) CH_4 and b) H_2O activation on Ni(111) and Cu(111).

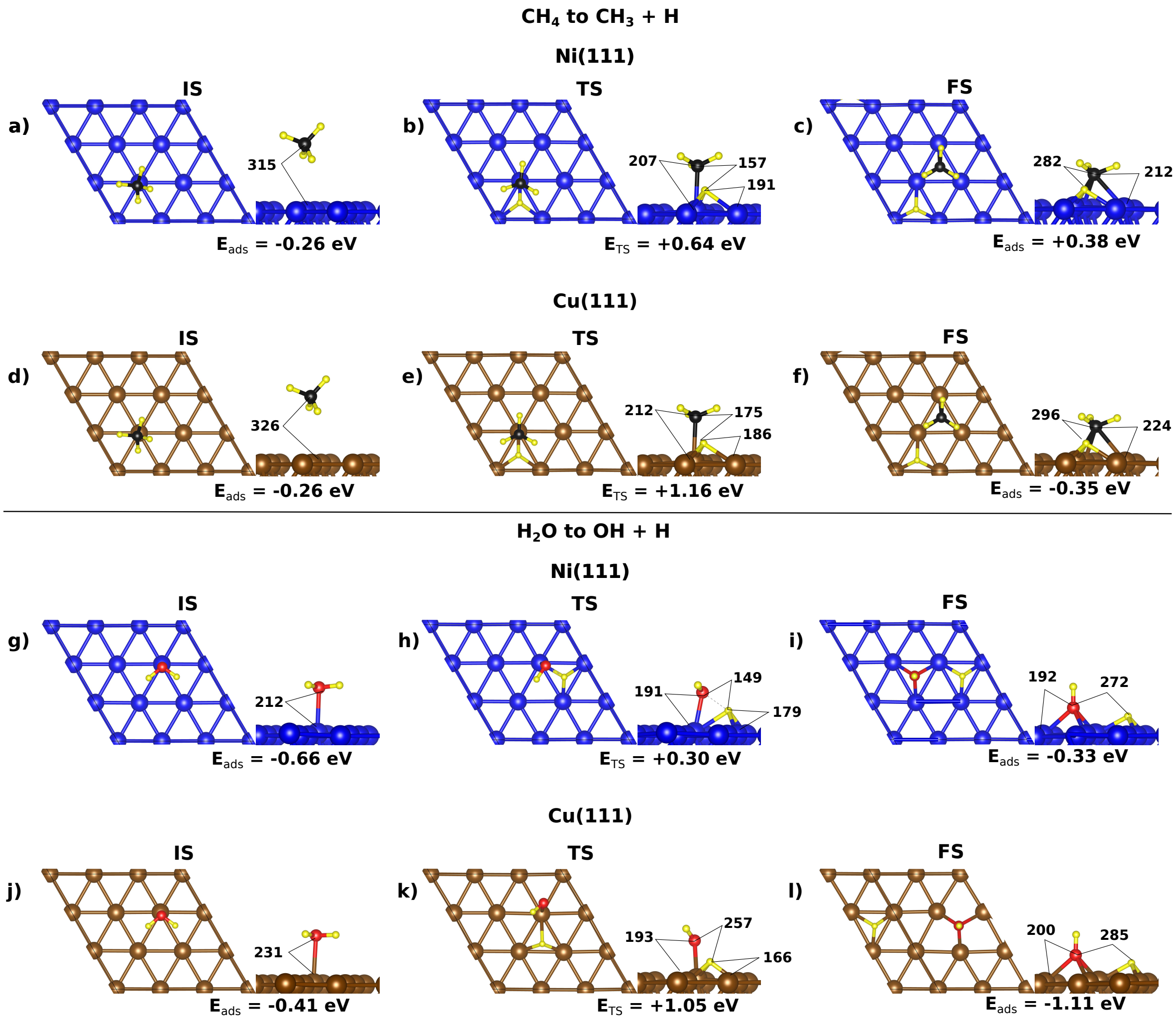

Figure S3: Initial, transition and final state structures for the first dehydrogenation of CH_4 and H_2O on the Ni(111) and Cu(111) surfaces. Selected distances are indicated in pm.

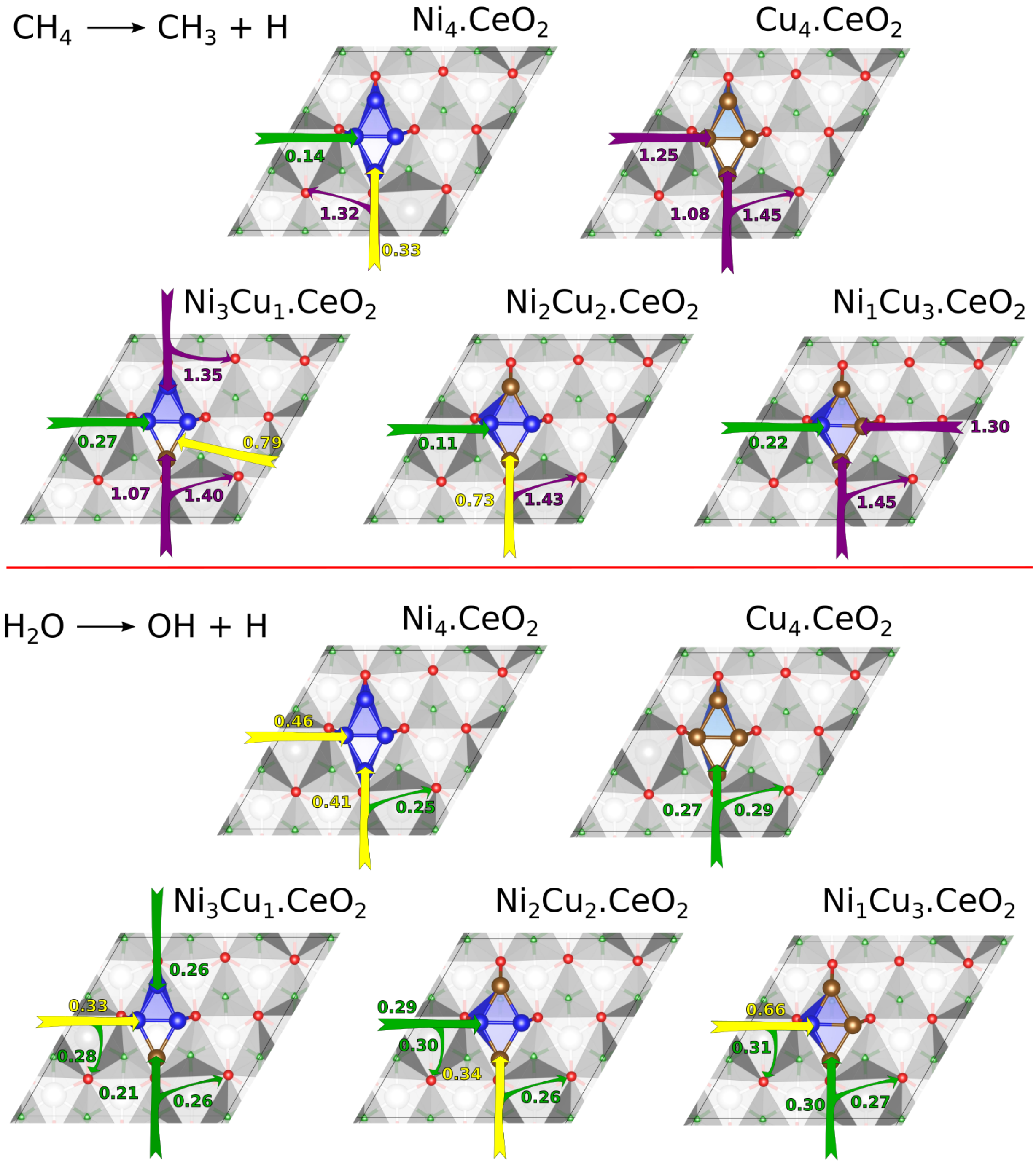

Figure S4: Energy barriers for the dissociation of CH_4 (upper panel) and H_2O (lower panel) for each $Ni_{4-x}Cu_x.CeO_2$ model catalyst. The thick arrows indicate the activation sites of the $CH_4 \rightarrow CH_3 + H$ reaction pathways. For pathways where a Ni or Cu site in the $Ni_{4-x}Cu_x$ cluster and a surface O site work cooperatively, that is, in the final state, OH species are bound to the metal particle whereas H species form OH species with surface oxygen atoms, the thin arrows indicate the location of the H atom.

Table S4: Energy (in eV) and geometrical parameters for the molecular initial state (IS) structure of the adsorption of CH_4 on $Ni_{4-x}Cu_x.CeO_2$. Distances between the carbon atom and the bimetallic particle (C–B), as well as between the carbon atom and the hydrogen atoms (C–H), are indicated (in pm). The charge gained by the C atom upon adsorption of CH_4 with respect to molecule in the gas phase is also indicated. All energies are relative to CH_4 in the gas phase and the corresponding clean surfaces.

State	Activation Site	E_{IS} (eV)	d(C–B) (pm)	d(C–H) (pm)	$q - q_{CH_{4gas}}$ (C)
			$CH_4/Ni_4.CeO_2$		
IS.1	Ni (at. 4)	–0.24	212	119; 110 (×3)	0.16
IS.2	Ni (at. 3)	–0.38	221	114; 112; 110 (×2)	0.11
			$CH_4/Ni_3Cu_1.CeO_2$		
IS.1	Ni (at. 4)	–0.10	270	111; 110 (×3)	0.08
IS.2	NiCu (at. 2 and 3)	–0.23	365; 373	110 (×4)	0.05
IS.3	Cu (at. 3)	–0.26	308	110 (×4)	0.00
			$CH_4/Ni_2Cu_2.CeO_2$		
IS.1	Ni (at. 4)	–0.28	260	112; 110 (×3)	0.05
IS.2	Cu (at. 3)	–0.23	310	110 (×4)	0.01
			$CH_4/Ni_1Cu_3.CeO_2$		
IS.1	Ni (at. 4)	–0.22	245	110; 113; 110 (×3)	0.02
IS.2	Cu (at. 2)	–0.32	304	110 (×4)	0.04
			$CH_4/Cu_4.CeO_2$		
IS.1	Cu (at. 3)	–0.24	261	110 (×2); 111 (× 2)	0.06
IS.2	Cu (at. 4)	–0.16	319	110 (×4)	0.02

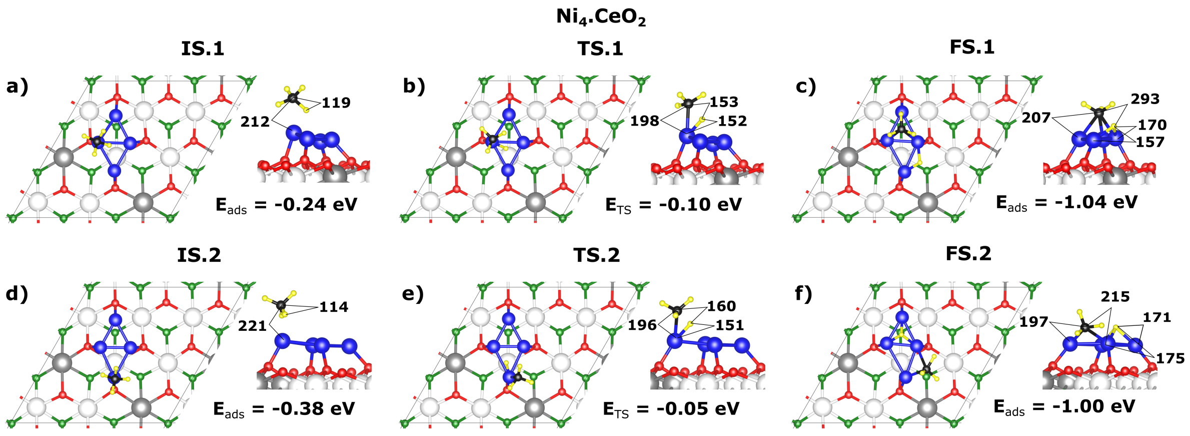

Figure S5: Initial, transition and final state structures for the non-cooperative first dehydrogenation of CH_4 on $Ni_4.CeO_2$ (cf. Figure S11). Representative distances are indicated in pm. Ni atoms are depicted in blue, while surface/subsurface oxygen atoms are in red/green, Ce^{4+} in white, and Ce^{3+} in gray.

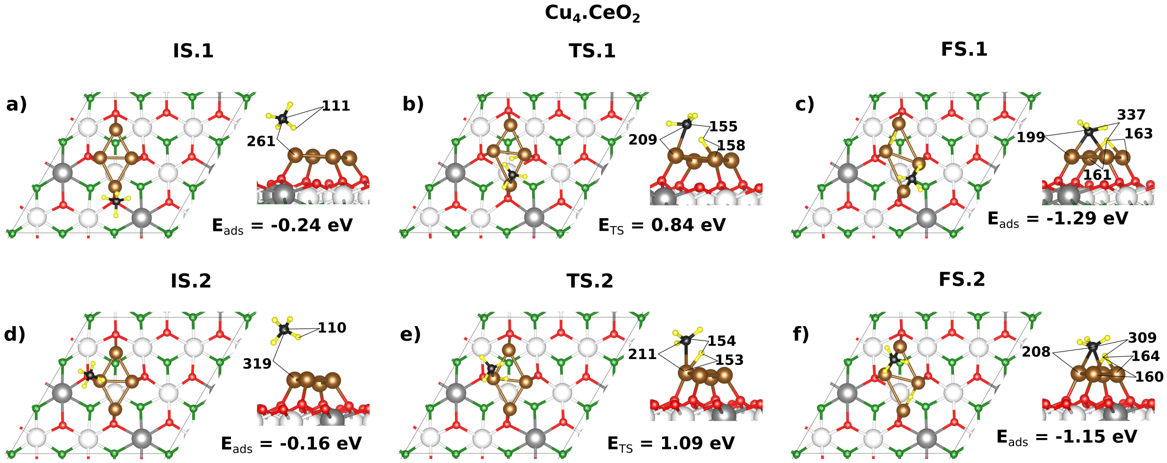

Figure S6: Initial, transition and final state structures for the non-cooperative first dehydrogenation of CH_4 on $Cu_4.CeO_2$ (cf. Figure S12). Representative distances are indicated in pm. Cu atoms are depicted in brown, while surface/subsurface oxygen atoms are in red/green, Ce^{4+} in white, and Ce^{3+} in gray.

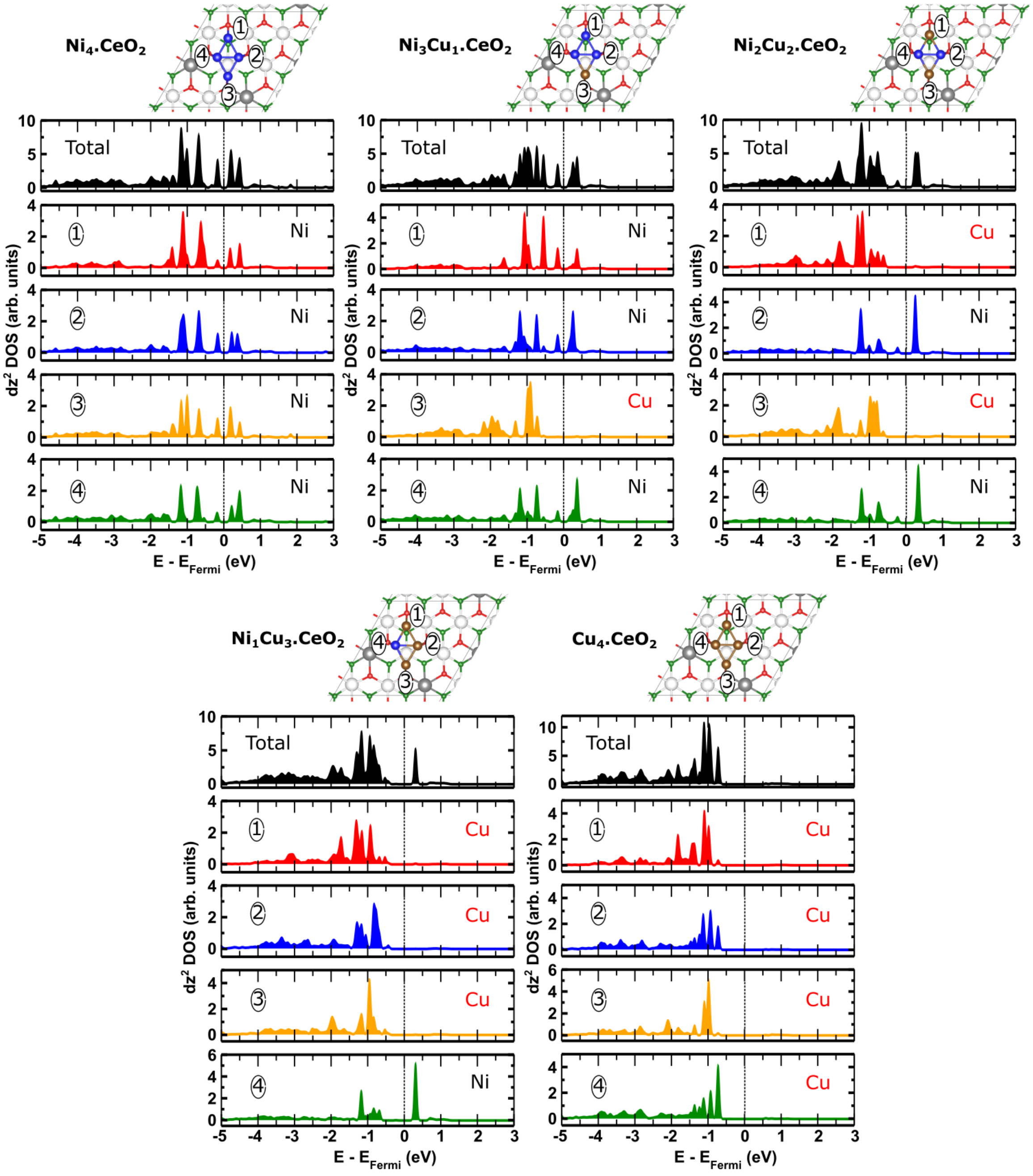

Figure S7: Total density of states (DOS) of the bimetallic particle and dz^2-projected density of states for each metallic atom in the particle.

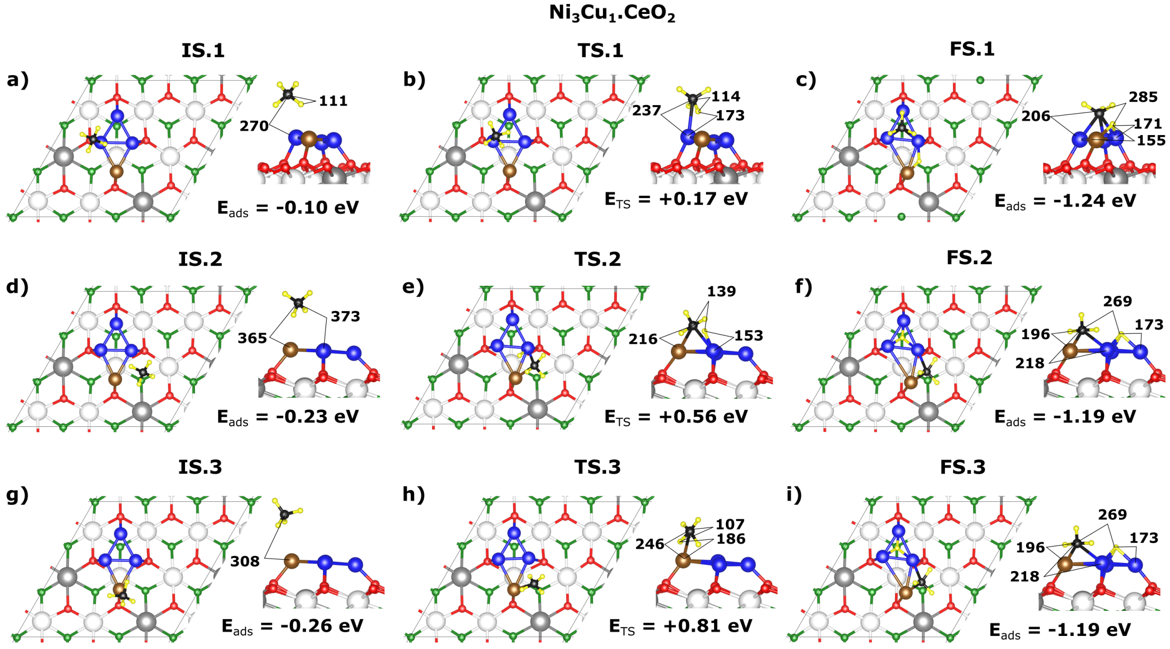

Figure S8: Initial, transition and final state structures for the non-cooperative first dehydrogenation of CH_4 on $Ni_3Cu_1.CeO_2$ (cf. Figure S13). Representative distances are indicated in pm. Ni and Cu atoms are depicted in blue and brown,respectively, while surface/subsurface oxygen atoms are in red/green, Ce^{4+} in white, and Ce^{3+} in gray.

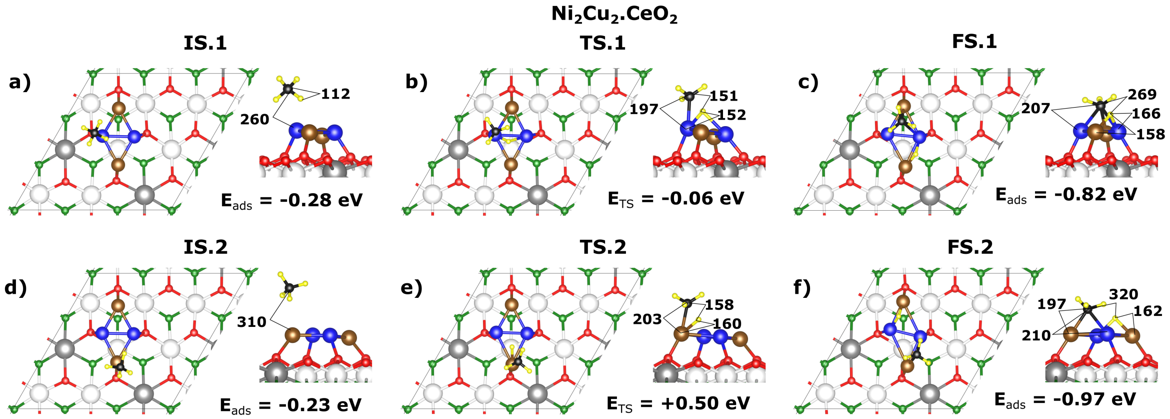

Figure S9: Initial, transition and final state structures for the non-cooperative first dehydrogenation of CH_4 on $Ni_2Cu_2.CeO_2$ (cf. Figure S14). Representative distances are indicated in pm. Ni and Cu atoms are depicted in blue and brown,respectively,while surface/subsurface oxygen atoms are in red/green, Ce^{4+} in white, and Ce^{3+} in gray.

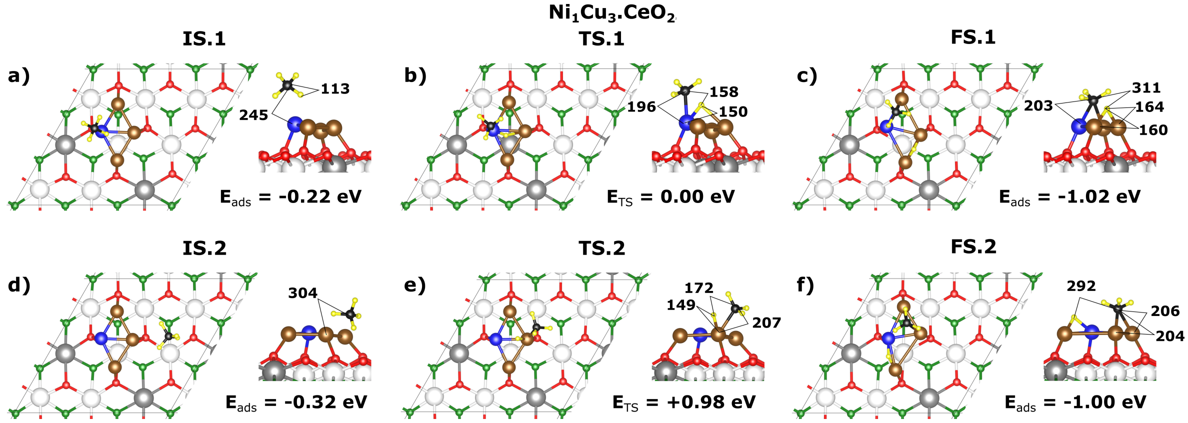

Figure S10: Initial, transition and final state structures for the non-cooperative first dehydrogenation of CH_4 on $Ni_1Cu_3.CeO_2$ (cf. Figure S15). Representative distances are indicated in pm. Ni and Cu atoms are depicted in blue and brown,respectively, while surface/subsurface oxygen atoms are in red/green, Ce^{4+} in white, and Ce^{3+} in gray.

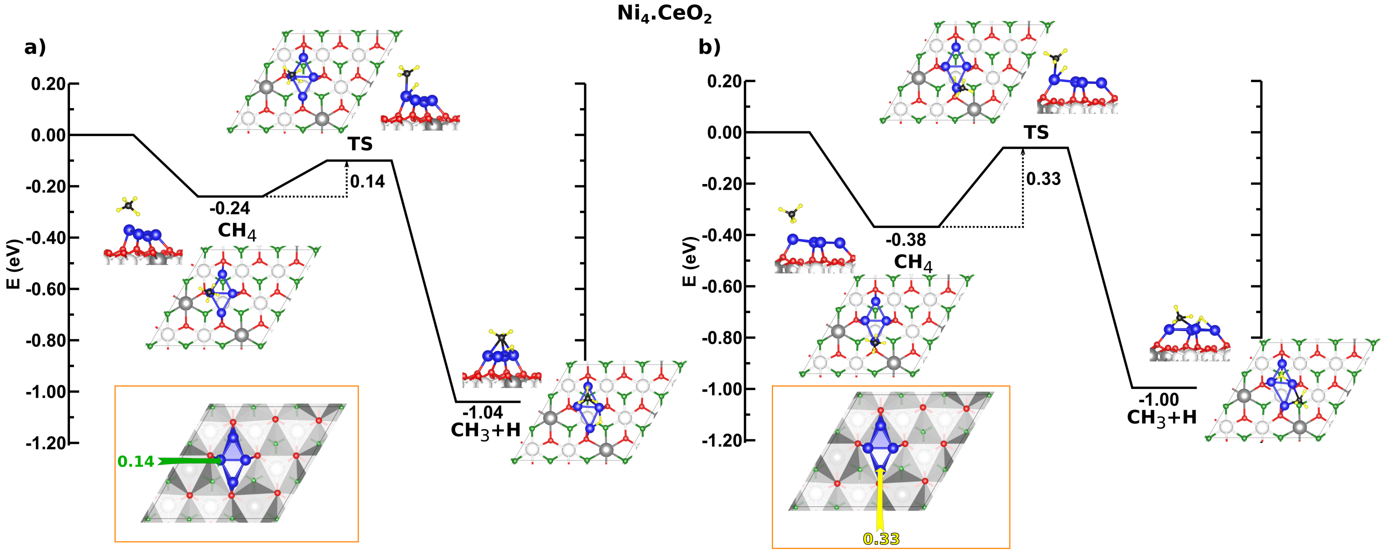

Figure S11: Non-cooperative reaction pathways for CH_4 activation on $Ni_4.CeO_2$.

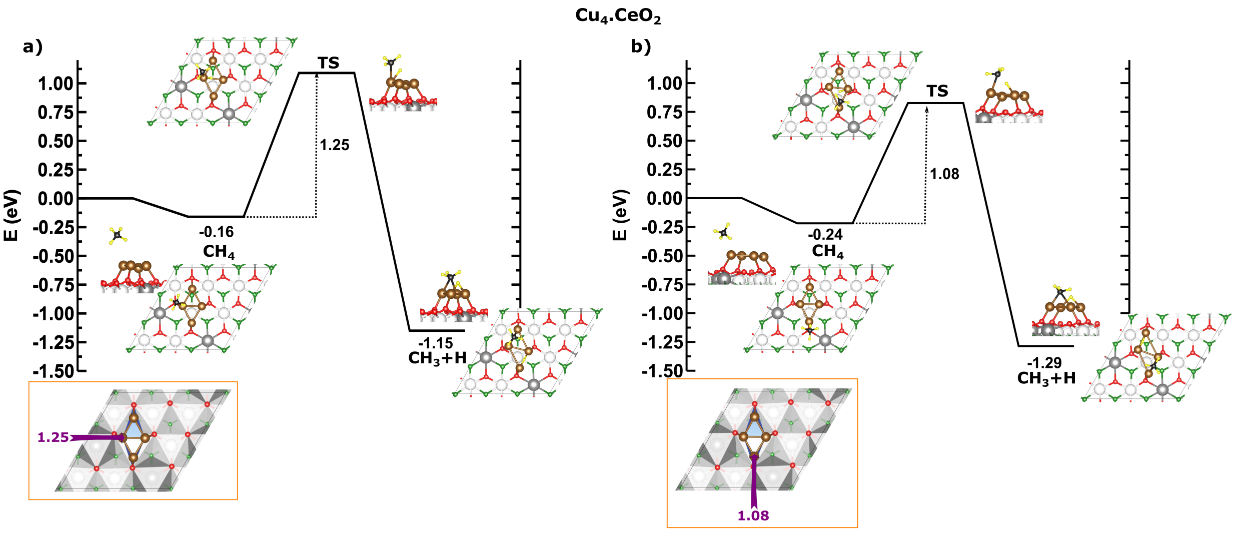

Figure S12: Non-cooperative reaction pathways for CH_4 activation on $Cu_4.CeO_2$.

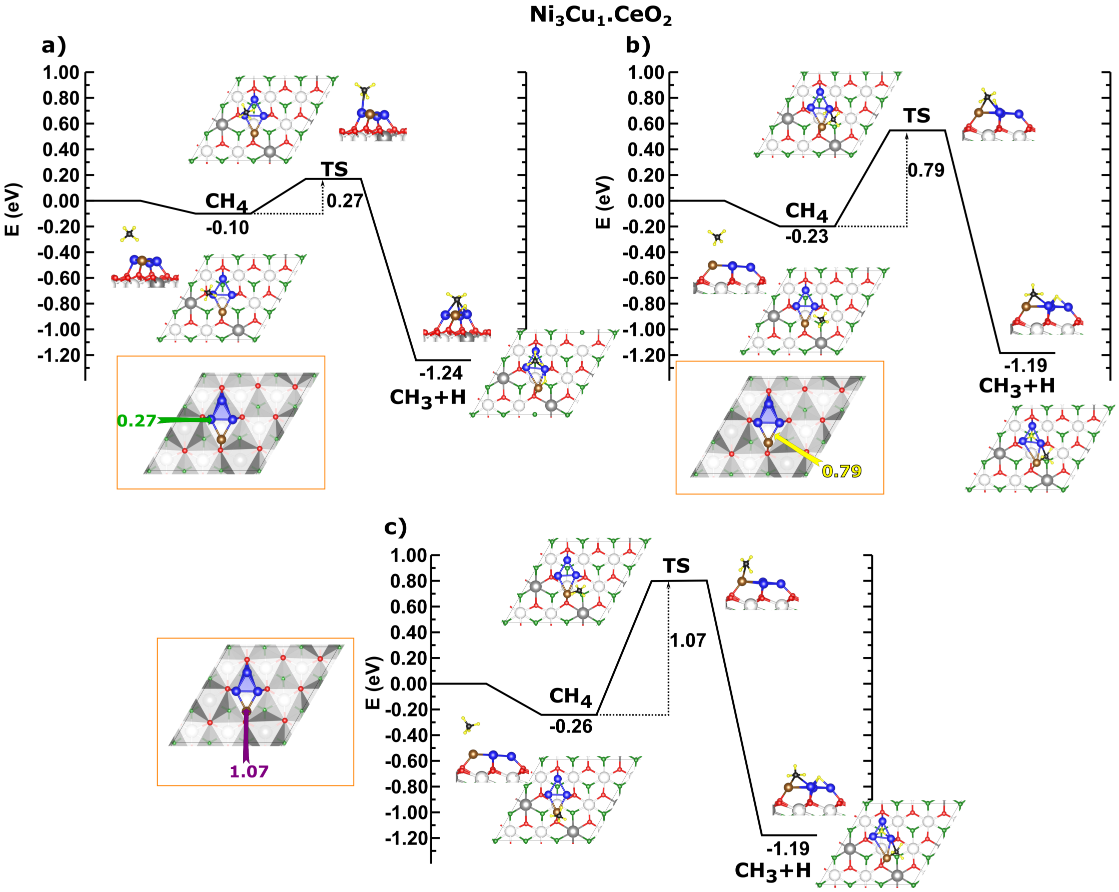

Figure S13: Non-cooperative reaction pathways for CH_4 activation on $Ni_3Cu_1.CeO_2$.

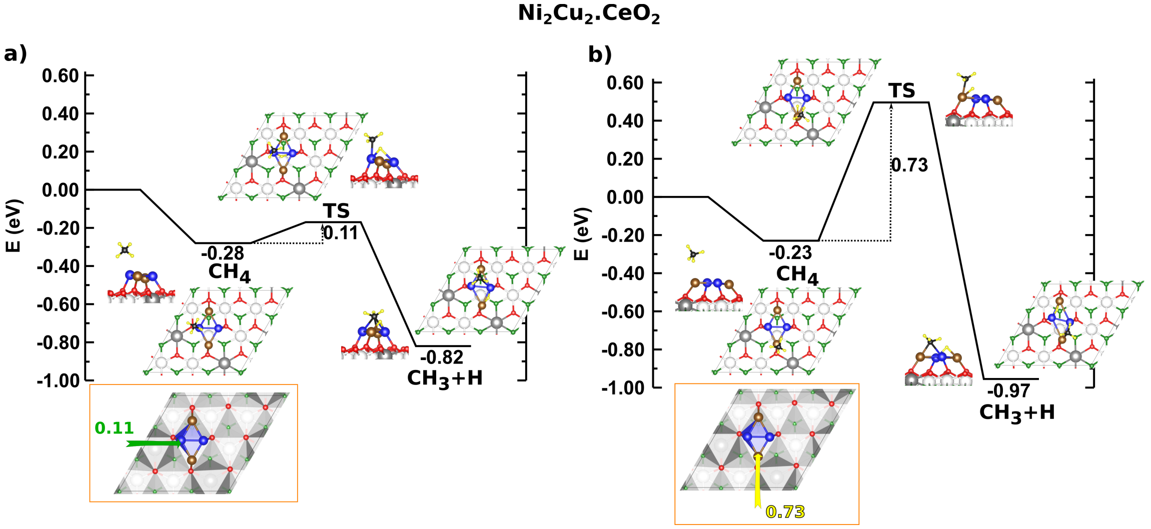

Figure S14: Non-cooperative reaction pathways for CH_4 activation on $Ni_2Cu_2.CeO_2$.

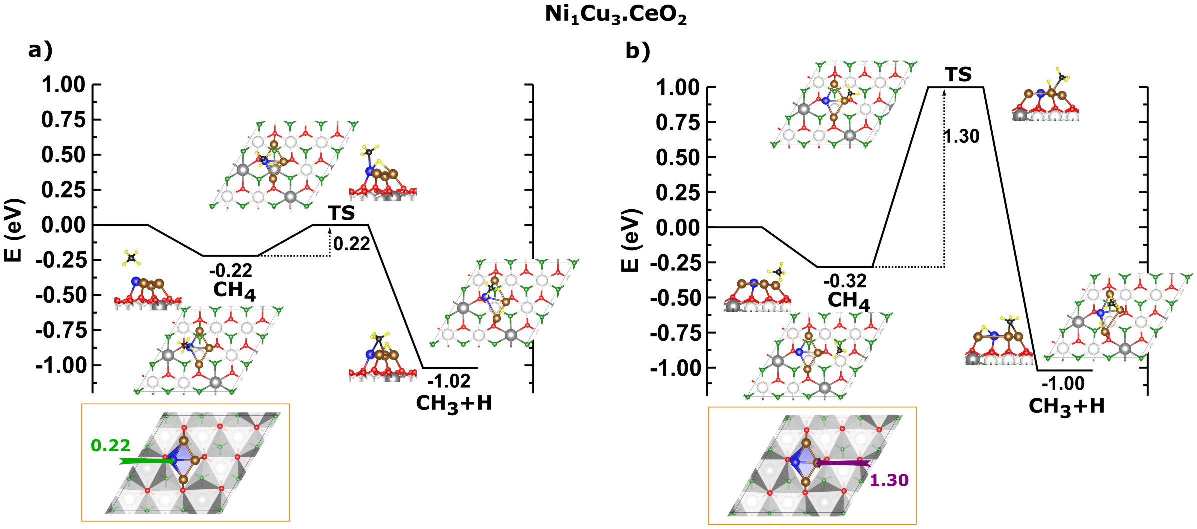

Figure S15: Non-cooperative reaction pathways for CH_4 activation on $Ni_1Cu_3.CeO_2$.

Table S5: Binding of the H atom to the supported clusters (in eV, with respect to $1/2$ H_2), calculated by removing the CH_3 species from the TS structures, with and without further optimization.

Hydrogen Adsorption Energy (eV)			
Catalyst	Full relax	TS single point	
		Cu Active Site	Ni Active Site
$Ni_4.CeO_2$	–1.22	–	–0.92
$Ni_3Cu_1.CeO_2$	–1.03	–0.55	–0.34
$Ni_2Cu_2.CeO_2$	–1.19	–0.72	–0.74
$Ni_1Cu_3.CeO_2$	–1.03	–0.50	–0.50
$Cu_4.CeO_2$	–0.60	–0.31	–

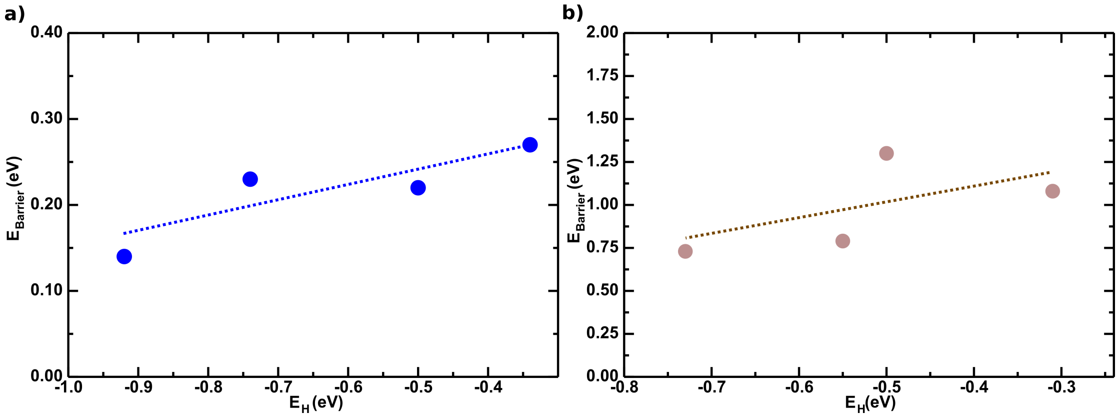

Figure S16: Energy barrier as a function of the H atom binding energy (with respect to $1/2$ H_2), calculated by removing the CH_3 species from the TS structures without further optimization, for a) Ni active site and b) Cu active site.

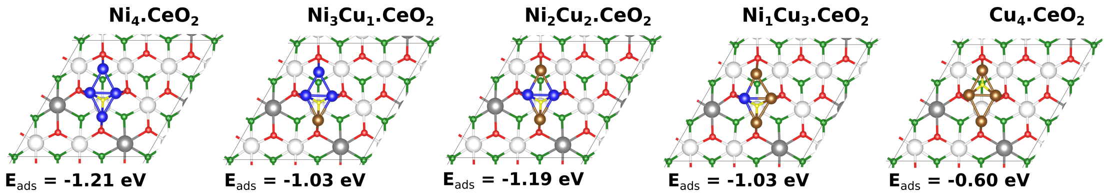

Figure S17: Hydrogen adsorption on the $Ni_{4-x}Cu_x.CeO_2$ surfaces, the binding energies were calculated respect to $1/2$ H_2.

Table S6: Energy (in eV) and geometrical parameters for the transition state (TS) structure of the $CH_4 \rightarrow CH_3 + H$ reaction over the $Ni_{4-x}Cu_x.CeO_2$ surfaces. Distances between the carbon atom and the bimetallic particle (C–B), as well as between the carbon atom and the hydrogen atoms (C–H), and between the Ni/Cu atoms and the hydrogen atoms (Ni/Cu–H), are indicated (in pm). All energies are relative to CH_4 in the gas phase and the corresponding clean surfaces.

**State**	**Activation Site**	**E_{TS} (eV)**	**d(C–B) (pm)**	**d(C–H) (pm)**	**d(Ni–H) (pm)**	**d(Cu–H) (pm)**
**$TS(CH_4 \rightarrow CH_3 + H)/Ni_4.CeO_2$**						
TS.1	Ni (at. 4)	–0.10	198	153<br>110 (×3)	152; 194<br>210; 292	–
TS.2	Ni (at. 3)	–0.05	196	160; 111<br>110 (×2)	151; 198<br>220; 353	–
**$TS(CH_4 \rightarrow CH_3 + H)/Ni_3Cu_1.CeO_2$**						
TS.1	Ni (at. 4)	+0.17	237	114<br>110 (×3)	173; 246<br>304	258
TS.2	NiCu (at. 2 and 3)	+0.56	216; 219	139<br>110 (×3)	153; 230<br>311	216
TS.3	Cu (at. 3)	+0.82	246; 293	107<br>110 (×3)	190; 290<br>368	186
**$TS(CH_4 \rightarrow CH_3 + H)/Ni_2Cu_2.CeO_2$**						
TS.1	Ni (at. 4)	–0.17	197	151<br>110 (×3)	152; 197	213; 295
TS.2	Cu (at. 3)	+0.50	203	158<br>110 (×3)	187; 194	160; 338
**$TS(CH_4 \rightarrow CH_3 + H)/Ni_1Cu_3.CeO_2$**						
TS.1	Ni (at. 4)	0.00	196	158<br>110 (×3)	150	199; 223<br>291
TS.2	Cu (at. 2)	+0.98	207	172<br>110 (×3)	213	149; 265<br>268
**$TS(CH_4 \rightarrow CH_3 + H)/Cu_4.CeO_2$**						
TS.1	Cu (at. 4)	+1.09	211	154<br>110 (×3)	–	153; 201<br>260; 264
TS.2	Cu (at. 3)	+0.84	209	155<br>110 (×3)	–	158; 200<br>222; 281

Table S7: Energy (in eV) and geometrical parameters for the final state (FS) structure of the $CH_4 \rightarrow CH_3 + H$ reaction over the $Ni_{4-x}Cu_x.CeO_2$ surfaces. Distances between the carbon atom and the bimetallic particle (C–B), as well as between the carbon atom and the hydrogen atoms (C–H), and between the Ni/Cu atoms and the hydrogen atoms (Ni/Cu–H), are indicated (in pm). All energies are relative to CH_4 in the gas phase and the corresponding clean surfaces.

**State**	**Activation Site**	**E_{FS} (eV)**	**d(C–B) (pm)**	**d(C–H) (pm)**	**d(Ni–H) (pm) (pm)**	**d(Cu–H) (pm)**
			**$(CH_3 + H)/Ni_4.CeO_2$**			
FS.1	Ni (at. 4)	–1.04	204; 207 216; 356	293; 114 111; 110	157; 170 303; 405	–
FS.2	Ni (at. 3)	–1.00	197; 215 372; 443	318; 113 110 (×2)	171; 175 (×2) 335	–
			**$(CH_3 + H)/Ni_3Cu_1.CeO_2$**			
FS.1	Ni (at. 4)	–1.24	206 (×2) 215; 372	285; 113 110 (×2)	171; 305 402	155
FS.2	NiCu (at. 2 and 3)	–1.19	196; 218 374; 440	313; 112 111; 110	170; 173 178	356
FS.3	Cu (at. 3)	–1.19	196; 218 374; 440	313; 112 111; 110	170; 173 178	356
			**$(CH_3 + H)/Ni_2Cu_2.CeO_2$**			
FS.1	Ni (at. 4)	–0.82	203; 207 236; 370	269; 113 111; 110	166 273	158 382
FS.2	Cu (at. 3)	–0.97	197; 210 323; 443	320; 112 110 (×2)	163 244	162 389
			**$(CH_3 + H)/Ni_1Cu_3.CeO_2$**			
FS.1	Ni (at. 4)	–1.02	198; 203 299; 412	311; 115 110 (×2)	284	160; 164 399
FS.2	Cu (at. 2)	–1.00	204; 206 236; 397	292; 112 110 (×2)	166	157; 315 404
			**$(CH_3 + H)/Cu_4.CeO_2$**			
FS.1	Cu (at. 4)	–1.15	199; 208 301; 419	309; 113 110 (×2)	–	160; 164 275; 397
FS.2	Cu (at. 3)	–1.29	199; 206 329; 455	337; 113 110 (×2)	–	161; 163 289; 417

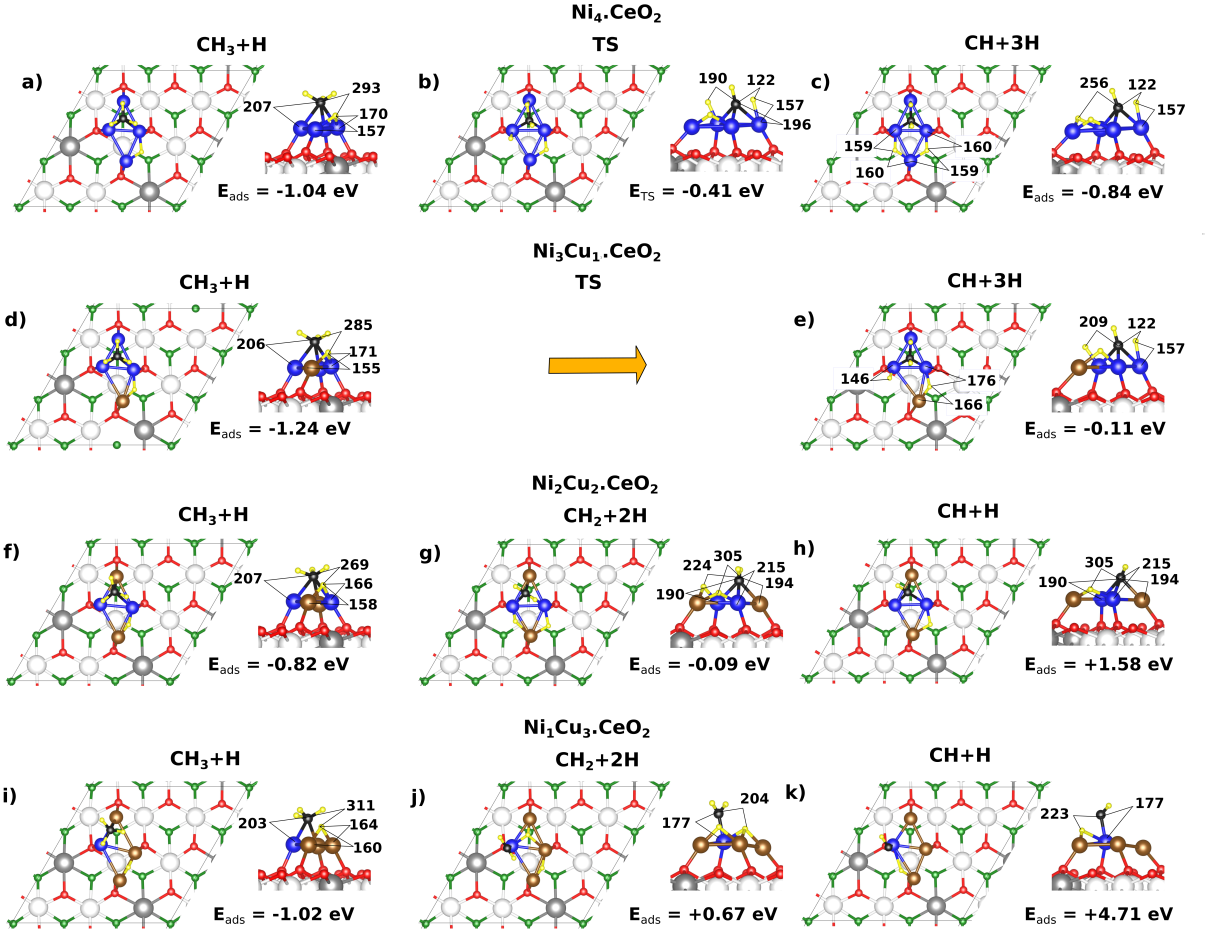

Figure S18: Intermediate states for the CH_4 dehydrogenation to CH + 3 H over the $Ni_{4-x}Cu_x.CeO_2$ surfaces, as reported in Figure 3 in the main text.

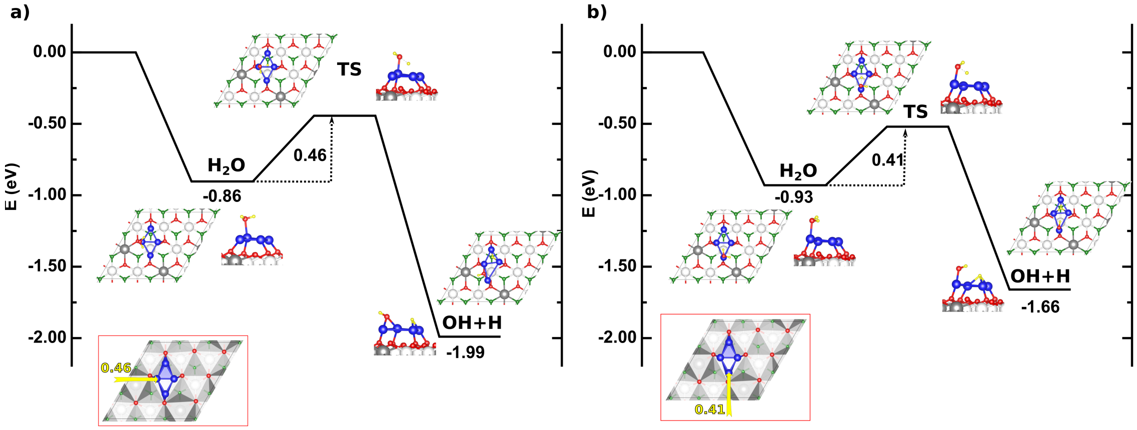

Figure S19: Non-cooperative reaction pathways for H_2O activation on $Ni_4.CeO_2$.

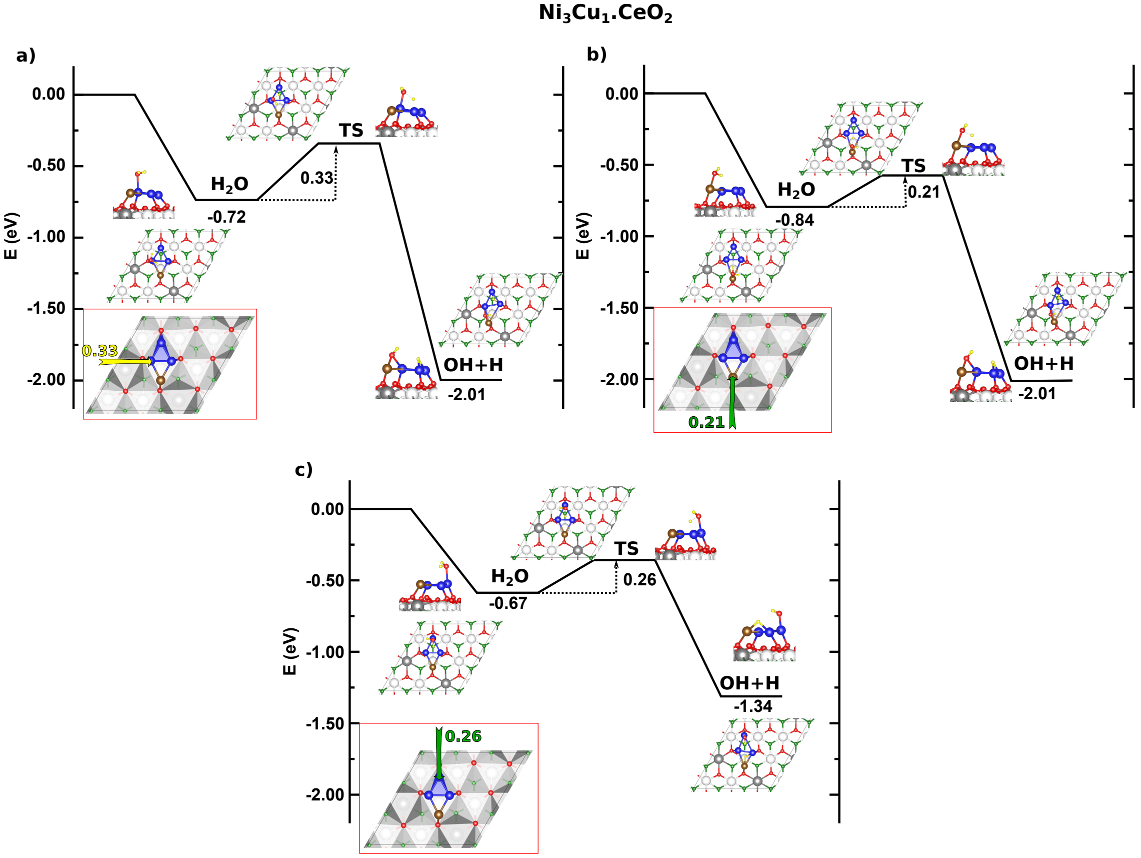

Figure S20: Non-cooperative reaction pathways for H_2O activation on $Ni_3Cu_1.CeO_2$.

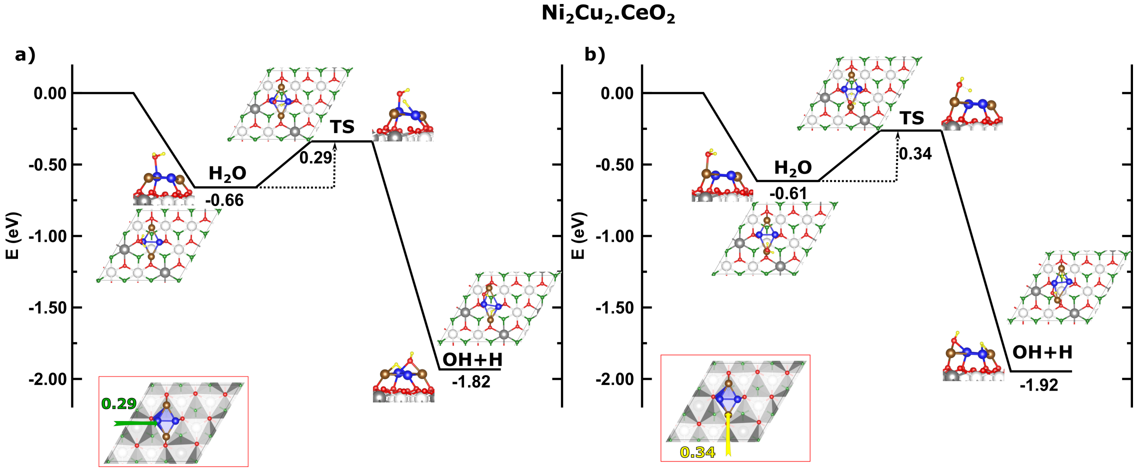

Figure S21: Non-cooperative reaction pathways for H_2O activation on $Ni_2Cu_2.CeO_2$.

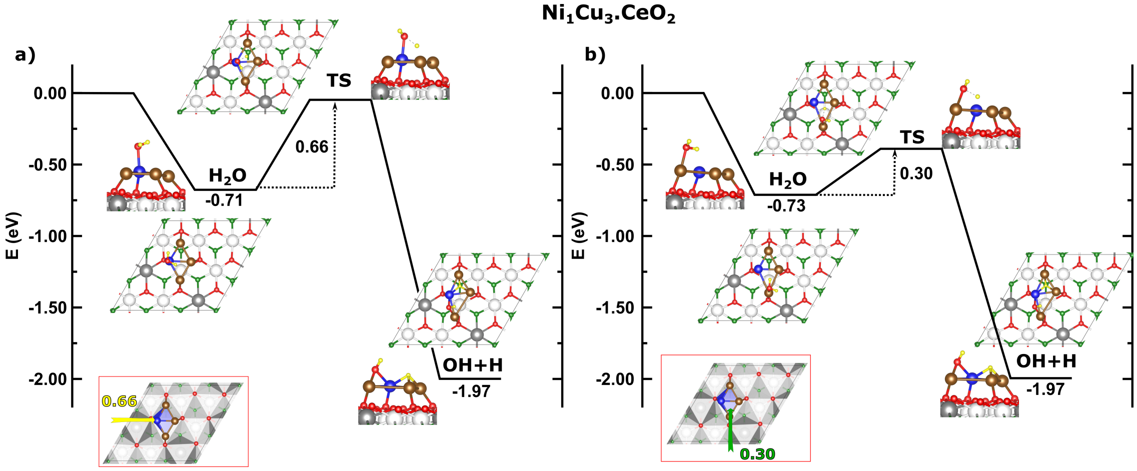

Figure S22: Non-cooperative reaction pathways for H_2O activation on $Ni_1Cu_3.CeO_2$.

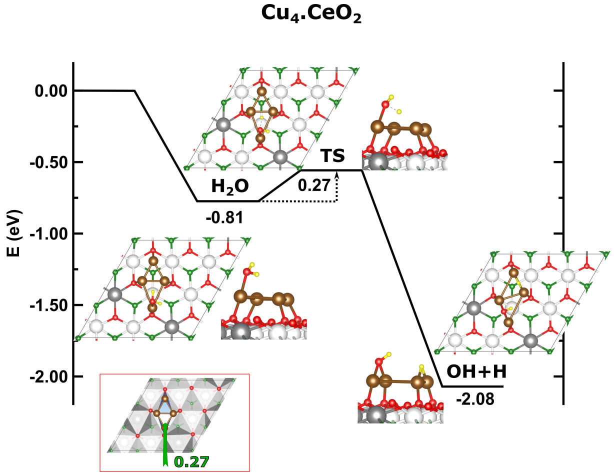

Figure S23: Non-cooperative reaction pathway for H_2O activation on $Cu_4.CeO_2$.

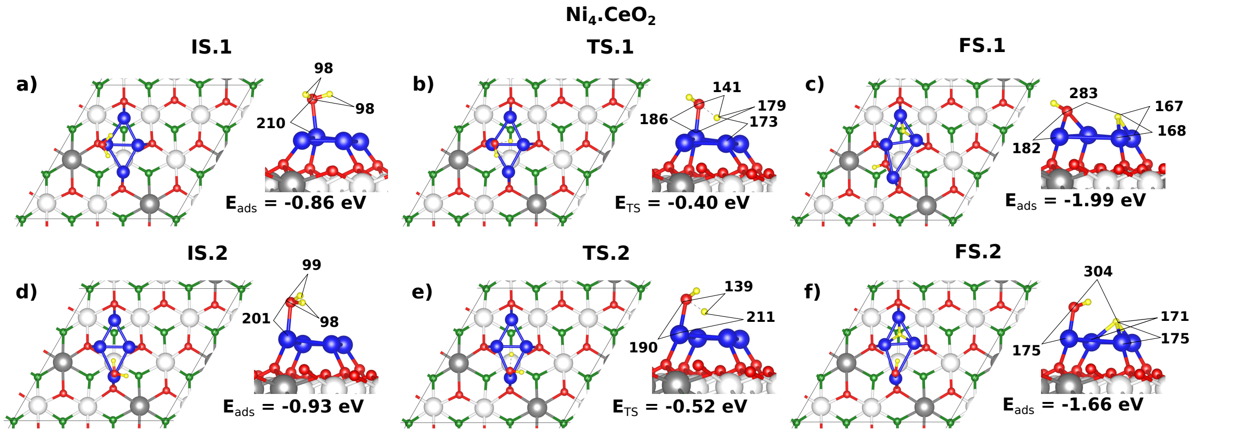

Figure S24: Initial, transition and final state structures for the non-cooperative first dehydrogenation of H_2O on $Ni_4.CeO_2$ (cf. Figure S19). Selected distances are indicated in pm. Ni atoms are depicted in blue, while surface/subsurface oxygen atoms are in red/green, Ce^{4+} in white, and Ce^{3+} in gray.

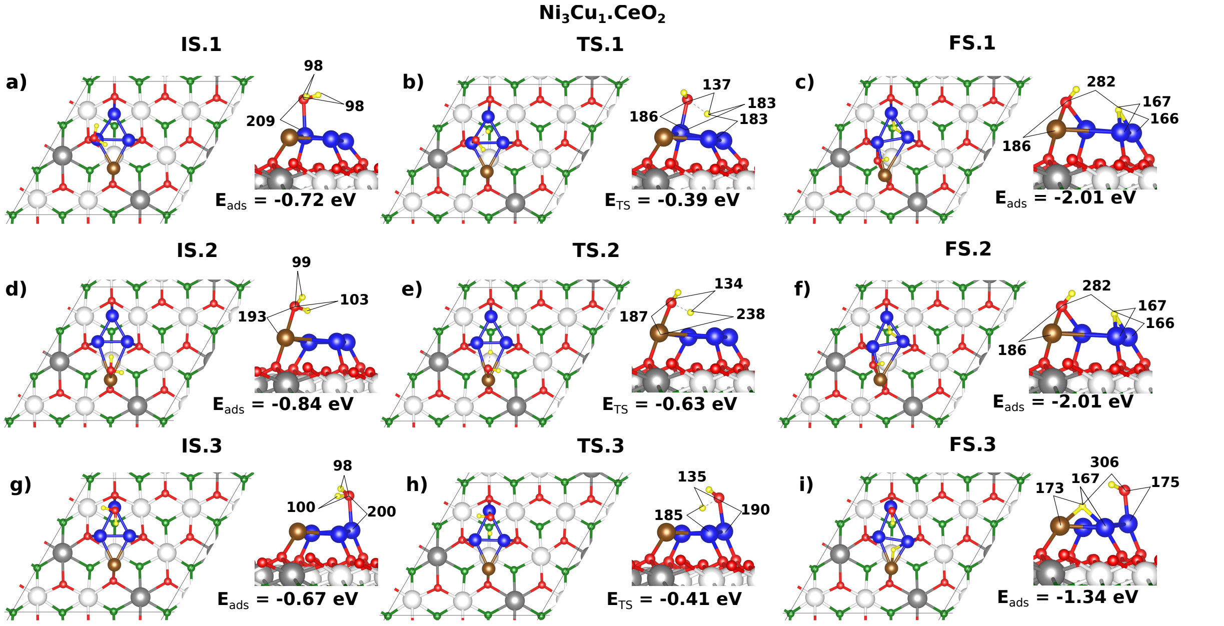

Figure S25: Initial, transition and final state structures for the non-cooperative first dehydrogenation of H_2O on $Ni_3Cu_1.CeO_2$ (cf. Figure S20). Selected distances are indicated in pm. Ni and Cu atoms are depicted in blue and brown,respectively, while surface/subsurface oxygen atoms are in red/green, Ce^{4+} in white, and Ce^{3+} in gray.

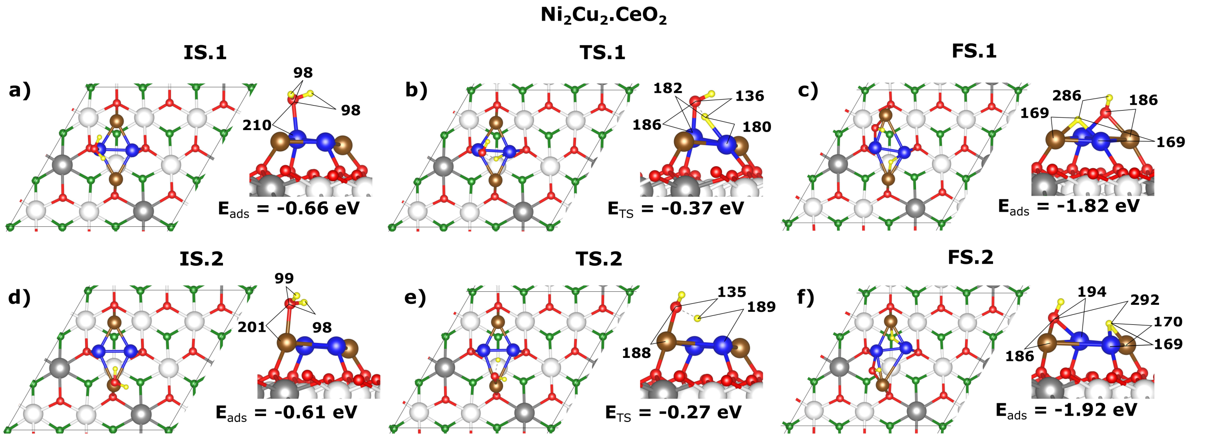

Figure S26: Initial, transition and final state structures for the non-cooperative first dehydrogenation of H_2O on $Ni_2Cu_2.CeO_2$ (cf. Figure S21). Selected distances are indicated in pm. Ni and Cu atoms are depicted in blue and brown,respectively,while surface/subsurface oxygen atoms are in red/green, Ce^{4+} in white, and Ce^{3+} in gray.

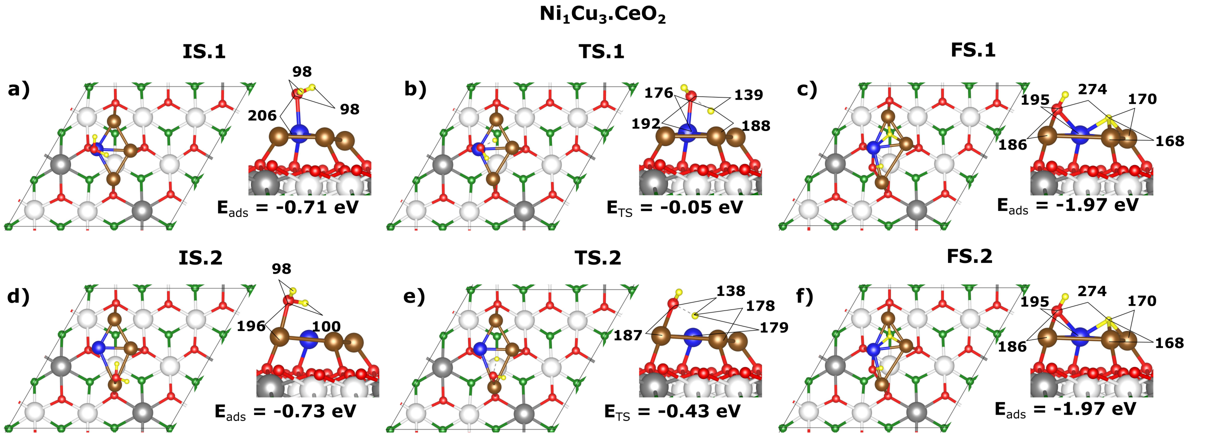

Figure S27: Initial, transition and final state structures for the non-cooperative first dehydrogenation of H_2O on $Ni_1Cu_3.CeO_2$ (cf. Figure S22). Selected distances are indicated in pm. Ni and Cu atoms are depicted in blue and brown,respectively, while surface/subsurface oxygen atoms are in red/green, Ce^{4+} in white, and Ce^{3+} in gray.

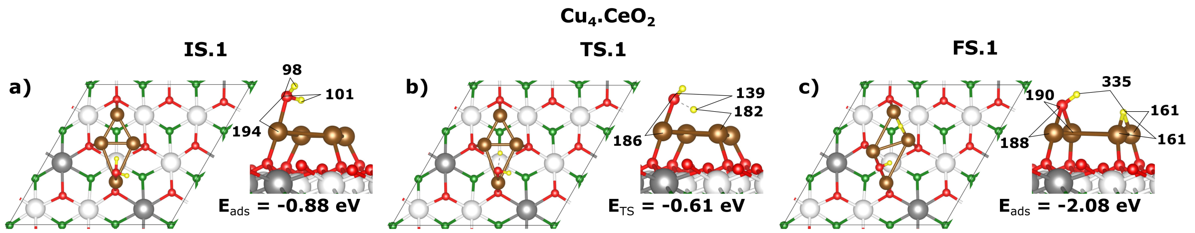

Figure S28: Initial, transition and final state structures for the non-cooperative first dehydrogenation of H_2O on $Cu_4.CeO_2$ (cf. Figure S23). Selected distances are indicated in pm. Cu atoms are depicted in brown, while surface/subsurface oxygen atoms are in red/green, Ce^{4+} in white, and Ce^{3+} in gray.

Table S8: Energy (in eV) and geometrical parameters for the molecular initial state (IS) structure of the adsorption of H_2O on $Ni_{4-x}Cu_x.CeO_2$. Distances between the oxygen atom in water and the bimetallic particle (C–A), as well as between the oxygen atom and the hydrogen atoms (O–H), are indicated (in pm). The charge gained by the O atom upon adsorption of H_2O with respect to molecule in the gas phase is also indicated. All energies are relative to H_2O in the gas phase and the corresponding clean surfaces.

State	E_{IS} (eV)	d(O–A) (pm)	d(O–H) (pm)	$q - q_{H_2O_{gas}}$ (O)
$H_2O/Ni_4.CeO_2$				
IS.1	−0.66	210	98 (×2)	−0.014
IS.2	−0.93	201	98; 99	−0.001
$H_2O/Ni_3Cu_1.CeO_2$				
IS.1	−0.62	209	98 (×2)	−0.006
IS.2	−0.84	193; 373	99; 103	0.05
IS.3	−0.67	200	98; 100	0.00
$H_2O/Ni_2Cu_2.CeO_2$				
IS.1	−0.66	210	98 (×2)	−0.020
IS.2	−0.61	203	98; 99	−0.017
$H_2O/Ni_1Cu_3.CeO_2$				
IS.1	−0.71	206	98 (×2)	−0.015
IS.2	−0.73	196	98; 100	+0.009
$H_2O/Cu_4.CeO_2$				
IS.1	−0.88	194	98; 101	+0.022

Table S9: Energy (in eV) and geometrical parameters for the transition state (TS) structure of the $H_2O \rightarrow OH + H$ reaction over the $Ni_{4-x}Cu_x.CeO_2$ surfaces. Distances between the oxygen atom and the bimetallic particle (O–B), as well as between the oxygen atom and the hydrogen atoms (O–H), and between the Ni/Cu atoms and the hydrogen atoms (Ni/Cu–H), are indicated (in pm). All energies are relative to H_2O in the gas phase and the corresponding clean surfaces.

State	E_{TS} (eV)	d(O–B) (pm)	d(O–H) (pm)	d(Ni–H) (pm)	d(Cu–H) (pm)
**$TS(H_2O \rightarrow OH + H)/Ni_4.CeO_2$**					
TS.1	–0.20	186	98; 141	173; 179; 229; 270	–
TS.2	–0.52	190	98; 139	185 (×2); 211; 396	–
**$TS(H_2O \rightarrow OH + H)/Ni_3Cu_1.CeO_2$**					
TS.1	–0.29	186	98; 137	183 (×2); 197	329
TS.2	–0.63	187	98; 134	182; 190; 301	238
TS.3	–0.41	190	98; 135	185; 186; 213	300
**$TS(H_2O \rightarrow OH + H)/Ni_2Cu_2.CeO_2$**					
TS.1	–0.37	186	98; 136	180; 182	200; 304
TS.2	–0.27	188	98; 135	189; 190	225; 318
**$TS(H_2O \rightarrow OH + H)/Ni_1Cu_3.CeO_2$**					
TS.1	–0.05	192	98; 139	176	188; 203; 308
TS.2	–0.43	187	98; 138	179	178; 229; 307
**$TS(H_2O \rightarrow OH + H)/Cu_4.CeO_2$**					
TS.1	–0.61	186; 277	98; 139	–	180; 182; 241; 302

Table S10: Energy (in eV) and geometrical parameters for the final state (FS) structure of the $H_2O \rightarrow OH + H$ reaction over the $Ni_{4-x}Cu_x.CeO_2$ surfaces. Distances between the oxygen atom and the bimetallic particle (O–B), as well as between the oxygen atom and the hydrogen atoms (O–H), and between the Ni/Cu atoms and the hydrogen atoms (Ni/Cu–H), are indicated (in pm). All energies are relative to H_2O in the gas phase and the corresponding clean surfaces.

State	E_{FS} (eV)	d(O–B) (pm)	d(O–H) (pm)	d(Ni–H) (pm)	d(Cu–H) (pm)
**$(OH + H)/Ni_4.CeO_2$**					
FS.1	–1.99	182; 194	98; 283	167; 168; 192; 374	–
FS.2	–1.66	175	98; 304	171 (×2); 175; 346	–
**$(OH + H)/Ni_3Cu_1.CeO_2$**					
FS.1/FS.2	–2.01	186; 197	97; 282	166; 167; 198	379
FS.3	–1.34	175	98; 306	167; 183; 338	173
**$(OH + H)/Ni_2Cu_2.CeO_2$**					
FS.1	–1.82	186; 195	97; 286	169; 185	167; 372
FS.2	–1.97	186; 194	97; 292	169; 180	170; 378
**$(OH + H)/Ni_1Cu_3.CeO_2$**					
FS.1/FS.2	–1.97	186; 195	97; 274	177	168; 170; 363
**$(OH + H)/Cu_4.CeO_2$**					
FS.1	–2.08	188; 190	98; 335	–	161 (×2)

References

(1) Kresse, G.; Furthmüller, J. Efficient Iterative Schemes for Ab Initio Total-Energy Calculations Using a Plane-Wave Basis Set. *Phys. Rev. B* **1996**, *54*, 11169–11186.

(2) Kresse, G.; Hafner, J. Ab initio Molecular Dynamics for Liquid Metals. *Phys. Rev. B* **1993**, *47*, 558–561.

(3) Kresse, G.; Joubert, D. From Ultrasoft Pseudopotentials to the Projector Augmented-Wave Method. *Phys. Rev. B* **1999**, *59*, 1758–1775.

(4) Dudarev, S. L.; Botton, G. A.; Savrasov, S. Y.; Humphreys, C. J.; Sutton, A. P. Electron-Energy-Loss spectra and the Structural Stability of Nickel Oxide: An LSDA+U Study. *Phys. Rev. B* **1998**, *57*, 1505–1509.

(5) Perdew, J. P.; Burke, K.; Ernzerhof, M. Generalized Gradient Approximation Made Simple. *Phys. Rev. Lett.* **1996**, *77*, 3865–3868.

(6) Grimme, S.; Antony, J.; Ehrlich, S.; Krieg, H. A consistent and Accurate Ab Initio Parametrization of Density Functional Dispersion Correction (DFT-D) for the 94 Elements H-Pu. *J. Chem. Phys.* **2010**, *132*, 154104.

(7) Grimme, S.; Ehrlich, S.; Goerigk, L. Effect of the Damping Function in Dispersion Corrected Density Functional Theory. *J. Comput. Chem.* **2011**, *32*, 1456–1465.

(8) Henkelman, G.; Uberuaga, B. P.; Jónsson, H. A Climbing Image Nudged Elastic Band Method for Finding Saddle Points and Minimum Energy Paths. *J. Chem. Phys.* **2000**, *113*, 9901–9904.

(9) Henkelman, G.; Jónsson, H. Improved Tangent Estimate in the Nudged Elastic Band Method for Finding Minimum Energy Paths and Saddle Points. *J. Chem. Phys.* **2000**, *113*, 9978–9985.

(10) Mao, Z.; Lustemberg, P. G.; Rumptz, J. R.; Ganduglia-Pirovano, M. V.; Campbell, C. T. Ni Nanoparticles on CeO2(111): Energetics, Electron Transfer, and Structure by Ni Adsorption Calorimetry, Spectroscopies, and Density Functional Theory. *ACS Catalysis* **2020**, *10*, 5101–5114.
